# A Comprehensive Overview of the Genes and Functions Required for Lettuce Infection by the Hemibiotrophic Phytopathogen Xanthomonas hortorum pv. *vitians*

**DOI:** 10.1128/msystems.01290-21

**Published:** 2022-03-21

**Authors:** Lucas Morinière, Laurène Mirabel, Erwan Gueguen, Franck Bertolla

**Affiliations:** a Université Lyon, Université Claude Bernard Lyon 1, CNRS, INRAE, VetAgro Sup, UMR Ecologie Microbienne, Villeurbanne, France; b Université Lyon, Université Claude Bernard Lyon 1, INSA, CNRS, UMR Microbiologie, Adaptation, Pathogénie, Villeurbanne, France; Oak Ridge National Laboratory; University of California, Berkeley

**Keywords:** *Xanthomonas hortorum*, phytopathogen, lettuce, Tn-seq, comparative genomics

## Abstract

The successful infection of a host plant by a phytopathogenic bacterium depends on a finely tuned molecular cross talk between the two partners. Thanks to transposon insertion sequencing techniques (Tn-seq), whole genomes can now be assessed to determine which genes are important for the fitness of several plant-associated bacteria *in planta*. Despite its agricultural relevance, the dynamic molecular interaction established between the foliar hemibiotrophic phytopathogen Xanthomonas hortorum pv. *vitians* and its host, lettuce (Lactuca sativa), remains completely unknown. To decipher the genes and functions mobilized by the pathogen throughout the infection process, we conducted a Tn-seq experiment in lettuce leaves to mimic the selective pressure occurring during natural infection. This genome-wide screening identified 170 genes whose disruption caused serious fitness defects in lettuce. A thorough examination of these genes using comparative genomics and gene set enrichment analyses highlighted that several functions and pathways were highly critical for the pathogen’s survival. Numerous genes involved in amino acid, nucleic acid, and exopolysaccharide biosynthesis were critical. The *xps* type II secretion system operon, a few TonB-dependent transporters involved in carbohydrate or siderophore scavenging, and multiple genes of the carbohydrate catabolism pathways were also critical, emphasizing the importance of nutrition systems in a nutrient-limited environment. Finally, several genes implied in camouflage from the plant immune system and resistance to immunity-induced oxidative stress were strongly involved in host colonization. As a whole, these results highlight some of the central metabolic pathways and cellular functions critical for *Xanthomonas* host adaptation and pathogenesis.

**IMPORTANCE** Xanthomonas hortorum was recently the subject of renewed interest, as several studies highlighted that its members were responsible for diseases in a wide range of plant species, including crops of agricultural relevance (e.g., tomato and carrot). Among *X. hortorum* variants, *X. hortorum* pv. *vitians* is a reemerging foliar hemibiotrophic phytopathogen responsible for severe outbreaks of bacterial leaf spot of lettuce all around the world. Despite recent findings, sustainable and practical means of disease control remain to be developed. Understanding the host-pathogen interaction from a molecular perspective is crucial to support these efforts. The genes and functions mobilized by *X. hortorum* pv. *vitians* during its interaction with lettuce had never been investigated. Our study sheds light on these processes by screening the whole pathogen genome for genes critical for its fitness during the infection process, using transposon insertion sequencing and comparative genomics.

## INTRODUCTION

Most Gram-negative bacterial plant pathogens behave as hemibiotrophs when interacting with their host plant (1). *Xanthomonas* species are indeed mostly considered hemibiotrophic pathogens that cause necrosis after a first asymptomatic phase (2–4). The infection process of a host plant by a nonvascular *Xanthomonas* is sequential: the pathogen displays different abilities in the cross talk with its host and ensures its own survival through nutrient acquisition systems and transformation of its microniche (5). *Xanthomonas* species initially start as epiphytes in the phyllosphere, an environment generally considered stressful and oligotrophic (6). There, in addition to deploying strategies to adhere to the leaf surface and survive abiotic stresses (7, 8), they rely on high-affinity TonB-dependent transporters (TBDTs) to take up simple sugars like sucrose and xylose (9, 10) or exploit more complex carbon sources (11). Biofilm formation at this stage participates in creating a safer and stable microenvironment for the population to develop (12).

The pathogen penetrates the leaves through natural openings (i.e., stomata, hydathodes, and wounds) and establishes itself within the apoplast to initiate its endophytic biotroph phase (5). It has to withstand the early basal responses triggered by the recognition of microbial-associated molecular patterns (MAMPs) by the plant pattern recognition receptors (PRRs) (13), feed on available nutrients, and synthesize compounds that are not directly available within its habitat. Polysaccharides such as cell-surface lipopolysaccharides (LPS) and the secreted exopolysaccharide (EPS) xanthan help to dampen the host immune response (14, 15). The type III secretion system (T3SS) also plays a major role in this phase, as it allows *Xanthomonas* species to disrupt the immune response signaling cascades and remodel the host metabolism to their own benefit through the intensive secretion of type III effectors (T3Es) (16, 17). Finally, the pathogen evolves toward a necrotrophic lifestyle: other cell death-inducing T3Es and the secretion of cell-wall-degrading enzymes (CWDEs) by the type II secretion system (T2SS) allow it to feed on leaked intracellular compounds and degraded cell components (1, 16).

Xanthomonas hortorum pv. *vitians* is a foliar pathogen of cultivated lettuce (Lactuca sativa); it causes a disease called bacterial leaf spot of lettuce (BLSL) (18), characterized by the progressive formation of small necrotic spots a few days after the pathogen has been inoculated. Then, these spots coalesce to form large necrotic patches that alter the quality and yield of the harvest without killing the entire plants (19, 20). BLSL has become a significant threat for lettuce producers over the past decades, and *X. hortorum* pv. *vitians* is considered a reemerging pathogen all around the world (18, 20–23). Nowadays, very little is known about the biology of this pathogen during the infection process, and the molecular bases sustaining the host-pathogen interaction remain mostly unelucidated (24).

To investigate the molecular biology and describe the essential genome of *X. hortorum* pv. v*itians in vitro*, a transposon insertion sequencing (Tn-seq) approach was recently developed for our model strain LM16734 (25). Tn-seq techniques rely on the generation of a saturated mutant library through large-scale transposon mutagenesis coupled with high-throughput next-generation sequencing and statistical analysis to estimate the essentiality and/or fitness contribution of each genetic feature in a selective environment (26). Tn-seq applications to bacteria of agricultural interest recently improved our understanding of the molecular cross talk between symbiotic or pathogenic bacteria and their host plants (27). For the plant-pathogenic species Pantoea stewartii subsp. *stewartii* (28), Dickeya dadantii (29), Pseudomonas syringae (30, 31), Ralstonia solanacearum (32), and Agrobacterium tumefaciens (33), Tn-seq provided an overall picture of the cellular functions and processes mobilized during plant infection and identified novel virulence factors.

We used our Tn-seq mutant library to screen for genes implied in lettuce colonization by *X. hortorum* pv. *vitians*. We applied a combination of comparative genomics, gene set enrichment analysis, and metabolic and cellular pathway mapping to draw biological sense from the list of genes defined as conditionally essential in lettuce. This approach successfully identified critical genetic features involved in amino acid and nucleic acid biosynthesis, CWDE secretion by the T2SS, carbohydrate nutrition and iron uptake, cellular polysaccharide synthesis, and oxidative stress resistance systems.

## RESULTS AND DISCUSSION

### Validation of the Tn-seq experimental design and assessment of bottleneck effects.

To investigate the fitness contribution of each genetic feature of *X. hortorum* pv. *vitians* LM16734 to growth in rich medium broth and during lettuce leaf infection, we used the previously developed and published Mariner-based Tn-seq mutant library (25). This library consists of at least 60,715 chromosomal mutants and 1,218 plasmidic mutants with transposon insertions at individual TA dinucleotide sites, out of a total of 85,314 chromosomal and 1,361 plasmidic TA sites within the *X. hortorum* pv. *vitians* LM16734 complete genome (5.23-Mb circular chromosome and 57-kb native plasmid pLM16734). The insertion densities in the library were ca. 71% and 89% on the chromosome and the plasmid, respectively.

First, the infection bottleneck effect arising from our inoculation procedure in young lettuce plants was estimated. The central paradigm behind Tn-seq experiments states that the mean read count per genetic feature is correlated with the corresponding mutant fitness in the tested selective environment (26). However, sampling bottleneck effects—which are stochastic events of loss of genetic diversity within a population when its size is drastically reduced (e.g., during host penetration and infection by a pathogen)—are known to blur data interpretation if not carefully considered in the experimental design (34, 35). Moreover, the selective DNA library preparation steps and random nature of mass sequencing can result in another type of bottleneck by reducing the genetic diversity within the extracted genomic library DNA (36). The number of generations experienced by the mutant library is also a critical parameter to be considered, as it directly impacts the fitness score calculated for each genetic feature (34, 37). Conceptually, a Tn-seq experiment consists of thousands of competition assays conducted simultaneously (34). Thus, variations in a mutant’s abundance caused by selective pressure will be correlated with the number of generations experienced by the population.

**FIG 1:**
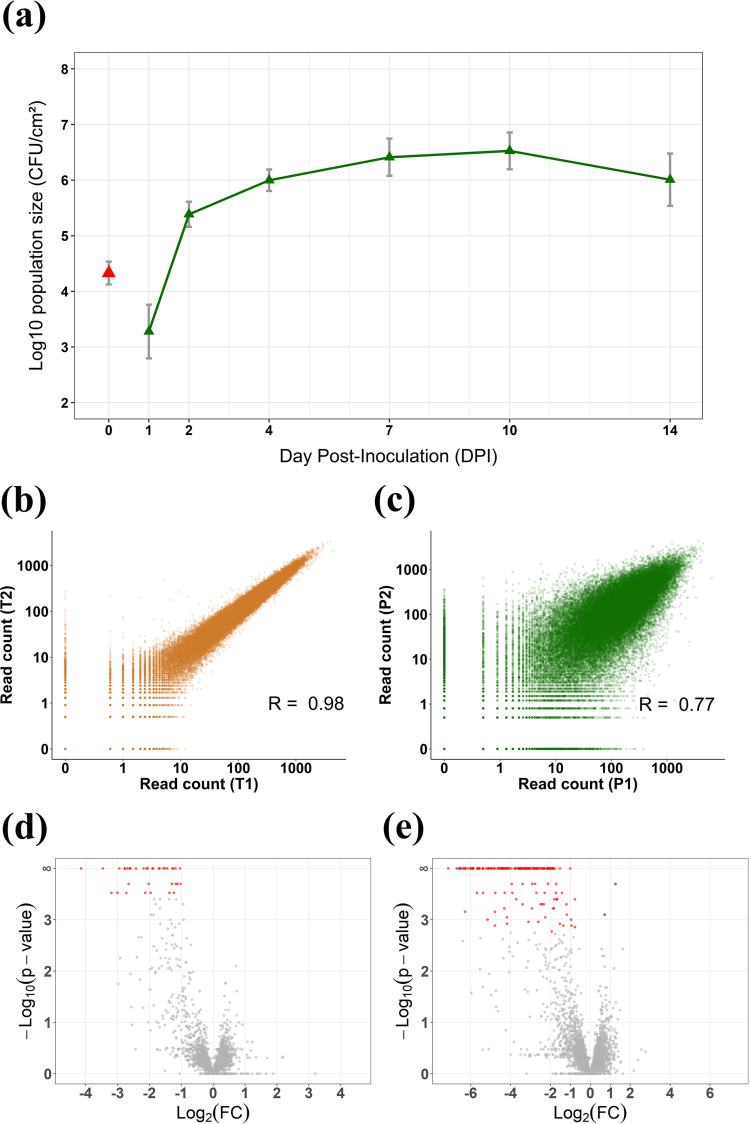
Validation of the experimental design and bottleneck assessment. (a) Evolution of the *X. hortorum* pv. *vitians* LM16734 population during lettuce infection, expressed as log_10_-transformed CFU · cm^−2^ of inoculated leaf. Red triangles, measured epiphytic populations following spray inoculation; green triangles, measured endophytic populations at each time point; gray bars, standard errors calculated on triplicates. (b and c) Read count correlation among Tn-seq duplicates for the *X. hortorum* pv. *vitians* LM16734 chromosome in (b) the inoculum and (c) lettuce leaves. (d and e) Volcano plots of the resampling results in (d) the inoculum versus the *in vitro* library condition and (e) lettuce leaves versus the inoculum condition. Plots represent the log_2_ fold change of each genetic feature as a function of the negative log_10_ of the resampling test *P* value. Genetic features that passed the FDR adjustment test (*q* value of ≤0.05) are indicated in red if they are conditionally essential and in blue if they provide a growth advantage. Nonsignificant genetic features are in gray.

A preliminary experiment was performed to estimate the number of bacterial cells spray-inoculated on lettuce leaves and the number of cells penetrating the leaves, as well as to monitor the population size over the course of infection. Approximately 2.5 × 10^4^ CFU · cm^−2^ on average were deposited on the leaves during inoculation, and 5 × 10^3^ CFU · cm^−2^ were measured inside the leaves after 24 h (Fig. 1a). Therefore, the theoretical successful infection ratio was 1:5, provided that no generation event occurred within this time interval. In reality, the founding population was likely to have been smaller than 5 × 10^3^ CFU · cm^−2^, as a few generations probably occurred inside the leaves during the first 24 h. The generation time of *X. hortorum* pv. *vitians* is ca. 4.5 h in rich medium *in vitro* (data not shown). The total foliar surface for 30 plants was estimated to be approximately 6,000 cm^2^, meaning that a total of 1.5 × 10^8^ CFU were inoculated on the leaves and that 3 × 10^7^ CFU were inside the leaves after 24 h. Regarding these estimations, a theoretical total mutant library coverage of 2,500× was inoculated, and a rough estimate of 50× to 500× maximum successfully invaded the plant tissue. Finally, the highest population level was achieved 10 days postinoculation (dpi), with an average of 5.6 × 10^6^ internal CFU · cm^−2^ (Fig. 1a). Theoretically, 10 bacterial generation events occurred between 1 and 10 dpi, without considering mortality events. In other comparable *in planta* Tn-seq experiments, similar numbers of generations proved to be adequate for efficient and unbiased fitness score assessment (30, 31, 38).

Read mapping statistics showed that 22 to 26 million reads mapped to unique TA sites in the inoculum duplicates (T1 and T2), and 26 to 30 million in the lettuce infection duplicates (P1 and P2) (Table 1). The insertion densities were 69.9 and 71.0% on the chromosome and 88.5 and 87.9% on pLM16734 under the inoculum condition and 61.9 and 64.5% on the chromosome and 71.9 and 77.3% on pLM16734 under the *in planta* condition. Almost all library mutants were still present in the inoculum, and ca. 90% chromosomal and 80% plasmidic mutants were recovered in lettuce after 10 days. Moreover, Pearson correlation coefficients of read counts at unique TA sites between duplicates were 0.98 and 0.97 for the chromosome and plasmid pLM16734 under the inoculum condition, respectively, and 0.77 and 0.84 under the lettuce condition (Fig. 1b and 1c). These sequencing statistics confirmed that our experimental design bypassed the experimental bottleneck and that the technical bottleneck resulting from the DNA library preparation steps and sequencing was negligible.

**TABLE 1:** Transposon-insertion sequencing statistics before TTR normalization

Replicate	Sequencing yield	No. of Tn end-containing reads	Replicon	Read count (no. of unique TA sites)	No. of TA hits^*a*^	Insertion density^*b*^	Median read count over non-zero TA^*c*^
T1	25,830,465	22,029,814	Chromosome	21,916,438	59,675	69.9	100.9
			pLM16734	1,262,371	1,205	88.5	62.6

T2	39,102,386	26,462,594	Chromosome	26,330,348	60,579	71.0	99.6
			pLM16734	956,994	1,196	87.9	65.6

P1	30,091,641	26,195,853	Chromosome	26,067,795	52,850	61.9	99.9
			pLM16734	1,227,568	979	71.9	77.3

P2	34,333,964	30,135,609	Chromosome	29,982,890	55,013	64.5	93.8
			pLM16734	1,236,398	1,052	77.3	70.05

a The *X. hortorum* pv. *vitians* LM16734 genome contains 85,314 chromosomal TA sites and 1,361 plasmidic TA sites.

b Insertion density reflects the percentage of TA sites with at least one read mapped over the total number of TA sites on the replicon.

c Mean read count per TA site containing at least one read.

### Identification of 170 critical genes *in planta* and overrepresentation of anabolic processes.

**FIG 2:**
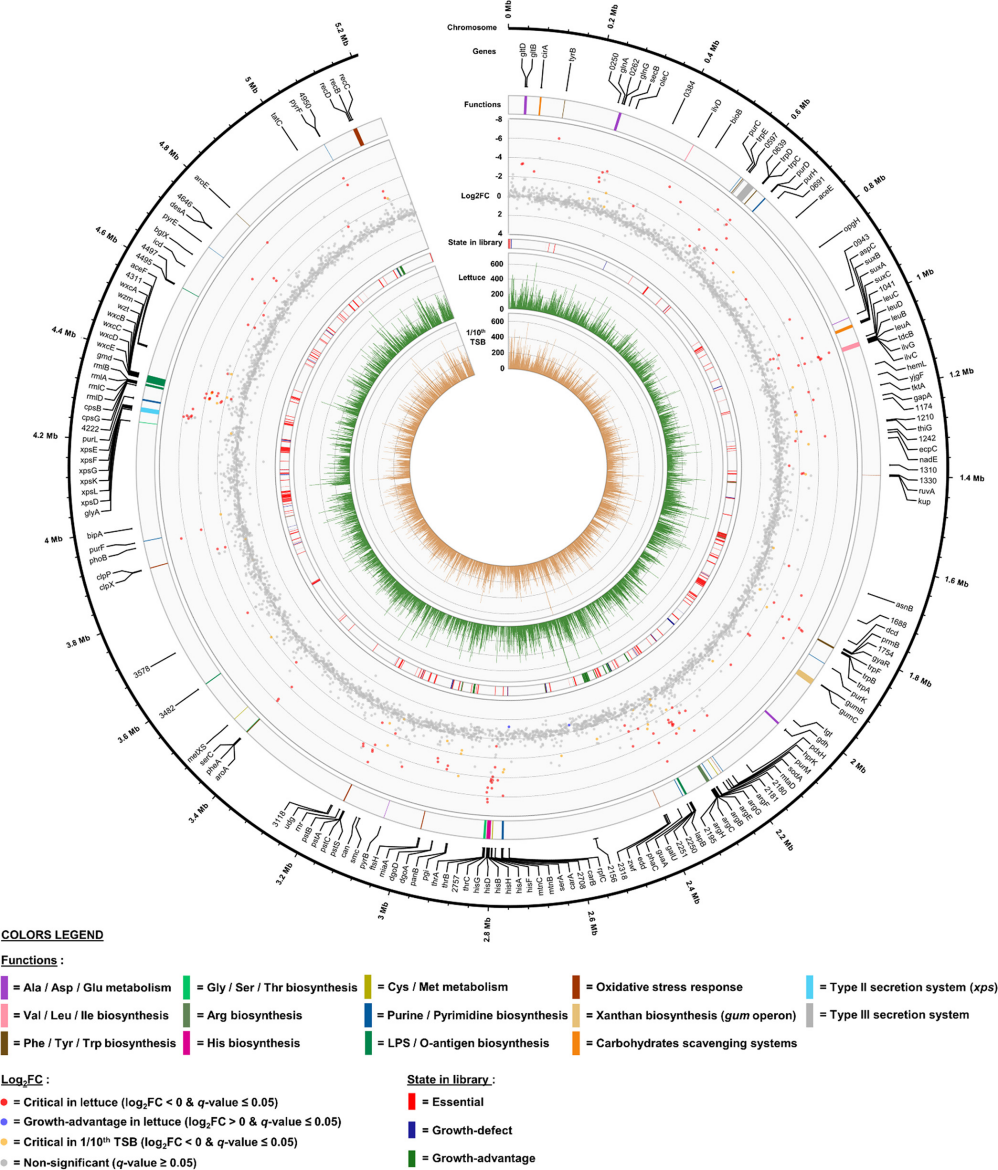
Genome atlas of the critical genetic features required for lettuce infection by *X. hortorum* pv. *vitians* LM16734. From the inner to outer track are the mean read count over 1 kb in 1/10^th^ TSB and in lettuce, the gene state in the *in vitro* library, as described in reference 24, the log_2_ FC score in lettuce leaves compared to the inoculum, clustering of critical genes, pathways, functions and structural components in lettuce, gene names or locus tag identifiers, and chromosomal tracks.

The resampling analyses conducted with TRANSIT identified 36 critical genes in 1/10^th^ tryptic soy broth (TSB) medium (Fig. 1d) and 170 critical genes and two growth advantage genes in lettuce (Fig. 1e). The complete gene lists and associated resampling metrics are available in Tables S1 to S4 in the supplemental material. There was a partial overlap between the two critical gene sets, as 29 genes in lettuce leaf were already considered critical in 1/10^th^ TSB. Additionally, we applied an extra filter by considering that mutants presenting a mean gene read count below 5 under the 1/10^th^ TSB condition showed decreased fitness *in vitro*, and 8 more genes were classified as critical in 1/10^th^ TSB. Examining the distribution of the critical genes in lettuce along the *X. hortorum* pv. *vitians* LM16734 chromosome revealed that a significant part seemed to be colocalized and organized in operons or gene clusters (Fig. 2).

10.1128/mSystems.01290-21.2 TABLE S1 Complete genome annotation and Tn-seq results *in vitro* and *in planta*. Download Table S1, XLSX file, 1.2 MB. Copyright © 2022 Morinière et al. 2022 Morinière et al. https://creativecommons.org/licenses/by/4.0/ This content is distributed under the terms of the Creative Commons Attribution 4.0 International license.

To determine which biological processes, pathways, and functions were overrepresented in our lettuce critical gene set, Gene Ontology (GO) terms (39) and KEGG ortholog (KO) (40) identifiers were assigned to all *X. hortorum* pv. *vitians* LM16734 genes. The GO term enrichment analysis indicated that 43 GO terms were significantly enriched in our gene set (Table 2). Among the 35 biological process-related enriched GO terms, nucleotide, amino acid, polysaccharide, carbohydrate, nitrogen, and phosphorus metabolism-associated terms were most abundant. Overall, biosynthetic processes seemed to be more represented than degradation processes. For example, 67.7% of genes related to “Nucleobase biosynthetic process” (GO:0046112) versus 54.5% for “Nucleobase metabolic process” (GO:0009112) and 42.5% of genes associated with “Cellular amino acid biosynthetic process” (GO:0008652) versus 34.9% for “Cellular amino acid metabolic process” (GO:0006520) were identified. Interestingly, 5 out of 7 genes for “Phosphate ion transport” (GO:0006817) were also critical *in planta*.

**TABLE 2:** Significantly enriched Gene Ontology terms in the critical genes in the lettuce leaf subset

GO identifier	GO term	Total no. of genes^*a*^	No. of genes in subset^*b*^	Enrichment score^*c*^	*q* value^*d*^
Biological processes					
General processes					
GO:0006082	Organic acid metabolic process	298	64	2.686	0.0
GO:0016053	Organic acid biosynthetic process	167	49	2.188	0.0
GO:0044283	Small molecule biosynthetic process	226	56	1.965	0.0
GO:1901362	Organic cyclic compound biosynthetic process	233	39	1.696	0.006043
GO:0018130	Heterocycle biosynthetic process	223	37	1.7	0.008476
GO:0019438	Aromatic compound biosynthetic process	199	38	1.984	0.000196
GO:0046176	Aldonic acid catabolic process	4	3	5.8	0.033252
GO:0040007	Growth	90	14	2.543	0.035375

Nucleotide metabolism					
GO:0052803	Imidazole-containing compound metabolic process	12	6	5.887	0.011874
GO:0034654	Nucleobase-containing compound biosynthetic process	138	22	2.025	0.013182
GO:0055086	Nucleobase-containing small molecule metabolic process	124	26	1.901	0.015897
GO:0042435	Indole-containing compound biosynthetic process	6	5	5.292	0.017839
GO:0046112	Nucleobase biosynthetic process	9	6	4.451	0.022403
GO:1901293	Nucleoside phosphate biosynthetic process	73	20	2.205	0.000229
GO:0042430	Indole-containing compound metabolic process	11	7	7.289	0.000771
GO:0009112	Nucleobase metabolic process	11	6	4.42	0.03223

Amino acid metabolism					
GO:0006520	Cellular amino acid metabolic process	166	53	3.702	0.0
GO:0008652	Cellular amino acid biosynthetic process	94	40	2.453	0.0
GO:0009072	Aromatic amino acid family metabolic process	24	9	3.338	0.027736
GO:0000105	Histidine biosynthetic process	8	6	3.642	0.035375

Polysaccharide metabolism					
GO:0000271	Polysaccharide biosynthetic process	23	8	7.75	3.8e−05
GO:0033692	Cellular polysaccharide biosynthetic process	19	7	8.4	7.7e−05
GO:0044264	Cellular polysaccharide metabolic process	26	8	7.754	0.000214
GO:0005976	Polysaccharide metabolic process	35	9	6.524	0.00034
GO:0008653	Lipopolysaccharide metabolic process	12	6	5.546	0.003351

Carbohydrate metabolism					
GO:1901135	Carbohydrate derivative metabolic process	178	29	1.95	0.008547
GO:0046394	Carboxylic acid biosynthetic process	167	49	1.346	0.009947
GO:0006757	ATP generation from ADP	15	4	4.938	0.028161

Nitrogen and phosphate metabolism					
GO:0019637	Organophosphate metabolic process	147	25	2.12	0.008476
GO:0044106	Cellular amine metabolic process	17	7	5.333	0.008547
GO:0019627	Urea metabolic process	4	4	8.25	0.009169
GO:0071941	Nitrogen cycle metabolic process	6	4	7.754	0.027141
GO:0000050	Urea cycle	4	4	5.515	0.028161
GO:0006793	Phosphorus metabolic process	244	34	1.602	0.042509
GO:0006817	Phosphate ion transport	7	5	2.5	0.04326


Molecular functions					
GO:0016829	Lyase activity	93	21	3.246	0.000101
GO:0016614	Oxidoreductase activity, acting on CH-OH group of donors	49	11	3.6	0.002604
GO:0016879	Ligase activity, forming carbon-nitrogen bonds	42	12	1.907	0.008476
GO:0048037	Cofactor binding	251	28	1.86	0.015897
GO:0005315	Inorganic phosphate transmembrane transporter activity	4	3	10.533	0.018301
GO:0016638	Oxidoreductase activity, acting on the CH-NH_2_ group of donors	8	4	8.333	0.024953
GO:0042802	Identical protein binding	144	19	1.661	0.033252
GO:0070279	Vitamin B6 binding	32	7	4.238	0.034385

a Total number of genes associated with the GO term in the *X. hortorum* pv. *vitians* LM16734 genome.

b Number of genes associated with the GO term in the subset of genes conditionally essential *in planta.*

c The simplified formula for the enrichment score (without pseudocounts) is log[(*b*/*q*)/(*m*/*p*)], where *b* is the number of genes with the GO term in the subset, *q* is the number of genes in the subset with a parent of the GO term, *m* is the total number of genes with the GO term in the genome, and *p* is the number of genes in the genome with a parent of the GO term. Thus, the enrichment score is the log-transformed ratio of the two relative abundances of genes with the GO term compared to those with a parent GO term (i) in the subset and (ii) in the complete genome. The calculation was derived from the Ontologizer method (114).

d Adjusted *P* value using the Benjamini-Hochberg false-discovery rate (FDR) test. GO terms with a *q* value of <0.05 were considered significantly enriched.

The KEGG pathway analysis reached more precision in the identification of the most critical metabolic and cellular pathways *in planta* (Table 3). For instance, it highlighted that even though a total of 49.5% of the genes involved in the *de novo* biosynthesis of amino acids were critical in lettuce, the relative proportions of genes varied among the specific amino acid pathways. The most impacted biosynthetic pathways were those of arginine (92.3% of the genes), valine, leucine, and isoleucine (76.9%), and alanine, aspartate, and glutamate (64%) biosynthesis. The genes involved in these pathways were often colocalized, like the *leu* and *ilv* genes encoding part of leucine and isoleucine biosynthesis or the *arg* and *his* operons for arginine and histidine, respectively (Fig. 2). Similarly, 19.6% of the carbon metabolism genes were critical, but the prevalent pathways were those of the 2-oxocarboxylic acid metabolism (57.1%), the pentose phosphate pathway (36%), the starch and sucrose metabolism (31.6%), and glycolysis/gluconeogenesis (29%). The genes related to the polysaccharide metabolism were mostly implied in O-antigen nucleotide sugar biosynthesis (56.3%). This analysis also pinpointed that 22.9% of the genes associated with the bacterial secretion systems were critical, which was not detected by the GO term enrichment analysis.

**TABLE 3:** Critical KEGG pathways *in planta* (nonexhaustive)

Pathway code	KEGG pathway	No. of genes:		% of genes in pathway
		Critical^*a*^	Total^*b*^	
General and diverse pathways				
xhr01110	Biosynthesis of secondary metabolites	84	301	27.9
xhr01240	Biosynthesis of cofactors	19	128	14.8
xhr00910	Nitrogen metabolism	9	14	64.3
xhr03070	Bacterial secretion system	16	70	22.9

Amino acid metabolism				
xhr01230	Biosynthesis of amino acids	54	109	49.5
xhr00220	Arginine biosynthesis	12	13	92.3
xhr00290	Valine, leucine, and isoleucine biosynthesis	10	13	76.9
xhr00250	Alanine, aspartate, and glutamate metabolism	16	25	64
xhr00400	Phenylalanine, tyrosine, and tryptophan biosynthesis	11	26	42.3
xhr00260	Glycine, serine, and threonine metabolism	14	35	40
xhr00340	Histidine metabolism	6	18	33.3
xhr00270	Cysteine and methionine metabolism	11	37	29.7

Carbon compound metabolism				
xhr01200	Carbon metabolism	18	92	19.6
xhr01210	2-Oxocarboxylic acid metabolism	12	21	57.1
xhr00030	Pentose phosphate pathway	9	25	36
xhr00500	Starch and sucrose metabolism	12	38	31.6
xhr00010	Glycolysis/gluconeogenesis	9	31	29
xhr00052	Galactose metabolism	5	20	25
xhr00051	Fructose and mannose metabolism	5	21	23.8

Nucleobase metabolism				
xhr00240	Pyrimidine metabolism	7	25	28
xhr00230	Purine metabolism	11	54	20.4

Polysaccharide metabolism				
xhr00541	O-antigen nucleotide sugar biosynthesis	9	16	56.3

a Number of genes identified as conditionally essential *in planta* (*q* value of ≤0.05).

b Total number of genes in model pathway in the *X. hortorum*-specific KEGG pathway database.

Overall, our results matched those of the other Tn-seq studies conducted on plant-pathogenic bacteria so far, in which many genes involved in the amino acid and nucleic acid metabolisms and in polysaccharide biosynthesis were critical *in planta* (28–30, 32). Moreover, random transposon mutant screening in other *Xanthomonas* species had already shown that mutating genes involved in these processes impaired growth and virulence *in planta* (41–44). For example, out of the 75 genes randomly screened as required for the virulence of Xanthomonas campestris pv. *campestris* in cabbage, 10 were involved in amino acid biosynthesis, 3 in *de novo* purine biosynthesis, 10 in lipopolysaccharide biosynthesis, and 3 in xanthan biosynthesis (41). This suggests that the central metabolic pathways required for plant colonization are equivalent across biological models and pathosystems, probably because plant-pathogenic bacteria face similar challenges in these selective environments: e.g., scarce nutrient or proteogenic amino acid availability.

Interestingly, no chemotaxis- or motility-related genes were screened as critical for the *in planta* lifestyle of *X. hortorum* pv. *vitians*, even though these features are usually considered important for pathogenicity and plant colonization (45). This could be explained by a combination of several factors. First, we hypothesized that during the spray-inoculation of our mutant library, some mutants may have been directly inoculated into the stomata and hydathodes. Thus, chemotaxis-driven motility was not required by these mutants to penetrate the leaves at the initial stages of plant infection. Second, motility might not be an indispensable feature for pathogenicity and *in planta* colonization by *Xanthomonas* species. A comparative genomic study of Xanthomonas fuscans subsp. *fuscans* suggested that the absence of motility could occur in field populations of several *Xanthomonas* species and that nonmotile variants would not undergo fitness defects in mixed populations, including flagellated strains (46). Furthermore, a new transcriptomic analysis of X. campestris pv. *campestris* conducted during cauliflower hydathode colonization revealed that the expression of most chemotaxis and motility genes was strongly repressed during the first days of infection (4). A similar observation was made during early rice infection by Xanthomonas oryzae pv. *oryzicola* (47). Such repression could even confer a stealth advantage to the bacterial population by reducing the production of microbial-associated molecular pattern (MAMP) molecules such as flagellar or pilus proteins (4, 46). Finally, chemotaxis and motility genes were not detected in the *in planta* RB-Tn-seq study of P. syringae, neither in the epiphytic screening nor in the apoplastic one (30). The *in planta* transcriptome analysis of Pseudomonas syringae infecting Arabidopsis thaliana also demonstrated that these genes were downregulated during plant infection (48), consistent with the fact that plant-pathogenic *Xanthomonas* and Pseudomonas species share similar overall infection strategies.

### Nutrient acquisition and assimilation as key features of the *in planta* lifestyle.

Numerous mutants of the carbohydrate metabolism were also strongly impaired in their fitness *in planta* (Tables 2 and 3). Interestingly, many genes of the central carbohydrate catabolism pathways—glycolysis and the pentose phosphate and Entner-Doudoroff pathways—were critical in lettuce, while none had been found indispensable on a glucose-rich medium (25). This time, most of the genes in these three pathways were conditionally essential either in lettuce or in 1/10^th^ TSB medium (see Fig. S1 in the supplemental material), and so were some key genes of the starch and sucrose degradation and fructose and mannose degradation pathways (Table 3). Unfortunately, no information is available about the chemical composition of the lettuce leaf apoplast or surface. Sucrose and malate are generally the most abundant carbohydrates in the apoplast of eudicots (49–52). In any case, this apparent metabolic versatility most probably contributes to the pathogen’s ability to adapt to the lettuce leaf environment, both outside and inside the leaf.

10.1128/mSystems.01290-21.1 FIG S1 Critical genes in the carbon metabolism pathways of *X. hortorum* pv. *vitians* LM16734 under the different Tn-seq experimental conditions, created with KEGG Mapper. Carbon numbers in compounds are indicated in circles or by asterisks for cofactors. Download FIG S1, TIF file, 2.6 MB. Copyright © 2022 Morinière et al. 2022 Morinière et al. https://creativecommons.org/licenses/by/4.0/ This content is distributed under the terms of the Creative Commons Attribution 4.0 International license.

**FIG 3:**
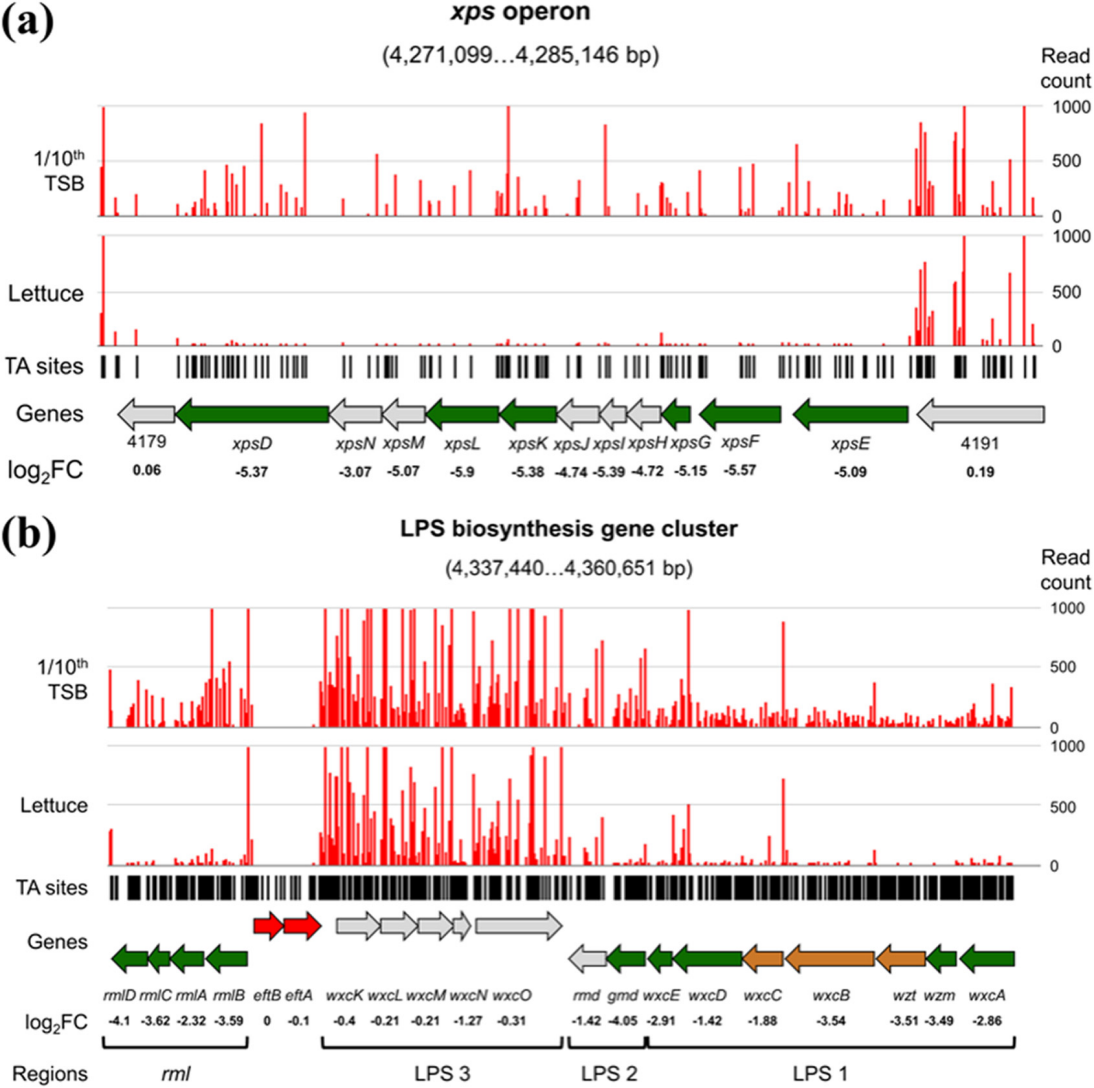
Schematic representation of (a) the critical genes in lettuce within the *xps* T2SS operon and (b) the LPS biosynthesis gene cluster of *X. hortorum* pv. *vitians* LM16734. Numbers in brackets indicate the width of the viewing window on the LM16734 genome. Green arrows, critical genes in lettuce; gold arrows, critical genes in 1/10th TSB; red arrows, essential genes in the *in vitro* library; gray arrows, nonsignificant genes. Gene names, log_2_ FC values, and—for panel b—grouping in functional regions as described in reference 67 are displayed below. Black bars, localizations of TA sites; bar plots, read counts at each TA site under the control and experimental conditions. (Values of >1,000 are not displayed.)

Consistent with numerous reports showing that *xps*-deficient *Xanthomonas* species mutants present reduced virulence *in planta* (41, 43, 44, 53), most of the *xps* T2SS mutants (XHV734_4180 to -4190) were positively screened as fitness deficient in our experiment (Fig. 3a). The *xps* genes that did not pass the significance threshold still displayed very low log_2_ fold change (FC) values (Table S1), suggesting that, despite their rejection by the statistical test, they were also critical in lettuce. Like the *X. hortorum* pv. *gardneri* genome (54), the *X. hortorum* pv. *vitians* LM16734 genome contained a large repertoire of genes encoding CWDEs, including various pectinolytic, cellulolytic, and hemicellulolytic enzymes. These genes were not critical in our analysis, either because of their high functional redundancy or because of potential complementation in *trans* by neighboring cells. Once the cell wall polymers have been degraded by the type II-secreted CWDEs, breakdown products and simple oligosaccharides have to be internalized by the pathogen to be assimilated. This role has been mainly attributed to TonB-dependent transporters (TBDTs) in *Xanthomonas* species (10). These TBDTs are primarily known for their role in iron-siderophore uptake and are often associated with carbohydrate sensor or degrading genes in so-called carbohydrate scavenging systems or carbohydrate utilization with TBDT (CUT) loci (10, 11). They allow for the scavenging of carbohydrates with high affinity and are thought to play an important role in bacterial adaptation to oligotrophic environments, such as plants for phytopathogens (9). Consequently, we searched for TBDT genes in our critical gene set and found three loci—XHV734_0066, -1016, and -1310—annotated as TonB-dependent receptors.

First, XHV734_1016 was located in the middle of a cluster of 3 critical genes (XHV734_1015 to -1017) (Fig. 2) which displayed a very strong homology and synteny with the *sux* CUT locus of X. campestris pv. *campestris*, responsible for sucrose-specific uptake and degradation (10). The *sux* gene cluster consists of *suxA* (XHV734_1016), coding for a sucrose TBDT that transports sucrose into the periplasm; *suxB* (XHV734_1015), coding for an intracellular amylosucrase that cleaves sucrose into glucose-1-P and fructose (55); *suxC* (XHV734_1017), coding for an inner membrane sucrose transporter; and *suxR* (XHV734_1018), coding for a LacI/GalR-family transcriptional repressor of sucrose import, which was logically not critical in our screening. The phosphohexose mutase encoded by *xanA* (XHV734_4228), which allows glucose-1-P to enter the glycolysis pathway, was also screened as critical. The conditional essentiality of this sucrose utilization locus in lettuce correlates with a previous study showing that sucrose was the primary carbon source of X. campestris pv. *campestris* in low-molecular-weight cabbage leaf extract during the first hours of incubation (56). Sucrose can also stimulate production of the diffusible signal factor (DSF) (57)—the *Xanthomonas* quorum-sensing signaling molecule that plays a major role in virulence regulation and survival *in planta* (58). Thus, sucrose may well be the preferential carbon source of *X. hortorum* pv. *vitians* during the early biotrophic phase of lettuce leaf invasion.

Second, XHV734_0066 was nearly identical to the *cirA* gene of *X. citri* subsp. *citri* 306, an oligoxyloside-specific TBDT involved in the uptake and utilization of xylan breakdown products (59). This *cirA* ortholog was adjacent to XHV734_0067, a xylose repressor-like transcriptional regulator strongly homologous to *xylR*, which regulates xylan degradation (9). Xylan from the cell wall hemicelluloses is mainly degraded into xylooligosaccharides by β-1,4-endoxylanases (60). *cirA* has been hypothesized to transport methylglucuronoxylotriose (MeGX_3_)—a tetrasaccharide intermediate of xylan degradation—into the periplasm, where it can be converted into xylotriose by a membrane-bound α-glucuronidase (59). There, xylotriose can be either broken down into d-xylose or directly transported to the cytoplasm. Once in the cytoplasm, d-xylose is converted into d-xylulose-5-P by XylA1, XylA2, and XylB (XHV734_2842, -4908, and -2843), processed by the pentose phosphate pathway (PPP), and finally assimilated (9). Intriguingly, the *tktA* gene encoding the transketolase of the PPP that catalyzes the conversion of d-xylulose-5-P into glyceraldehyde-3-P was critical for fitness, but the *xylA* and *xylB* genes were not. Therefore, their function could be redundant or performed by other enzymes. Xylan exploitation is important for *Xanthomonas* nutrition during the early phyllosphere colonization stage (9), but xylan could also be an important nutrient during the late necrotrophic phase when host cells are actively degraded.

Finally, XHV734_1310 carries a homolog of the ferric enterobactin receptor *bfeA* of various *Xanthomonas* spp., which is flanked by two other similar copies (XHV734_1309 and -1312) and located upstream of the xanthoferrin siderophore biosynthesis and uptake gene cluster *xss* (XHV734_1298 to -1305) (61). The organization of these three *bfeA*-like genes upstream of *xss* is strikingly similar to what was observed in *X. citri* subsp. *citri* (62) and *X. oryzae* pv *oryzae* (42). Wang et al. (42) named the genes *bfeA*, *bfeA2*, and *bfeA3* and reported that mutating *bfeA* alone ended up in a reduced-virulence phenotype *in planta*. Iron uptake using siderophores is indeed a well-known crucial feature of most bacterial phytopathogens necessary for successful host invasion (63). A recent proteomic analysis suggested that BfeA protein abundance could be directly correlated with virulence (64), which could partially explain why the gene is present in the form of three colocalized copies. However, further studies would have to elucidate why only one copy of *bfeA* is critical for fitness *in planta*.

### Hiding from the plant immune system using cellular polysaccharides.

The Tn-seq screening *in planta* highlighted a few critical genetic factors allowing *X. hortorum* pv. *vitians* to evade and counteract the lettuce immune response triggered by pathogen recognition during leaf invasion. The type III secretion system and its effectors are probably the best-known factors used by *Xanthomonas* to disrupt the host immune response (17). However, the genes involved in the T3SS of *X. hortorum* pv. *vitians* LM16734 (XHV734_0611 to -0633) did not pass the statistical significance threshold in our Tn-seq analysis, even though almost all of them presented a moderate fitness defect (Table S1). Only *hrpF* (XHV734_0597) was considered critical; it encodes the translocon required for T3E translocation into host cells. In comparable *in planta* Tn-seq experiments conducted in *D. dadantii* (29) and R. solanacearum (32), genes of the T3SS apparatus were not critical either because of the variable efficiency of Tn-seq screens in detecting genes involved in production and secretion of “public goods” (65). On the other hand, the statistically significant screening of T3SS genes in P. syringae was attributed to low bacterial apoplastic densities that did not allow complementation in *trans* (30). Thus, it seems that the genetic diversity of the transposon mutants coexisting in lettuce apoplastic spaces was high enough to observe a partial complementation phenomenon of the T3SS roles.

Following the identification of the cellular polysaccharide metabolism as significantly enriched in the GO term enrichment analysis (Table 2), we searched for genes associated with these processes in our critical gene set. As a result, two genes of the *gum* gene cluster encoding the synthesis of the EPS xanthan and 19 genes involved in the biosynthesis and assembly of cell surface LPS, clustered in 3 distinct genomic regions, were identified (Fig. 2). We already discussed the lethal phenotypes observed in our Tn-seq library for the *gumE*, *gumI*, and *gumJ* mutants (25), but here only *gumB* (XHV734_1853) and *gumC* (XHV734_1854) were critical in lettuce leaves. These two genes, along with *gumE*, are responsible for polymerization of the lipid-linked pentasaccharide units and subsequent export of the final xanthan EPS out of the periplasm (66). Moreover, xanthan production by *gumB* and *gumC* mutants of X. campestris was completely abolished *in vivo* (67). Xanthan gum is required for full epiphytic survival and virulence in *Xanthomonas* species (12, 42, 68).

The remaining 19 genes associated with LPS biosynthesis and assembly emphasized the crucial importance of these surface components in the interaction with the host plant. Three main gene clusters were identified (Fig. 2), each encoding specific functions. The first region contained 4 genes: the conserved LPS assembly gene *lapB* (XHV734_2249) (69), a homolog of the *rfb303* gene of *X. citri* subsp. *citri* (XHV734_2250) putatively associated with biosynthesis of the LPS core (70), a putative sugar epimerase gene (XHV734_2251), and the *galU* gene (XHV734_2252), which is involved in O-antigen nucleotide sugar biosynthesis (66, 71). Another small gene cluster consisting of XHV734_4495 and -4497 was related to LPS biosynthesis. XHV734_4497 is an O-antigen ligase-like enzyme putatively involved in ligating the O-antigen and the lipid A core.

The principal LPS gene cluster corresponded to the major *rml*/*wxc* cluster of LPS biosynthesis (72). Tn-seq screening distinguished which parts of this cluster were critical and consequently which particular functions of the LPS biosynthesis process were required for growth in lettuce (Fig. 3b). First, the *rmlBACD* critical genes (XHV734_4231 to -4233) encode a set of enzymes that convert glucose-1-phosphate into the nucleotide sugar dTDP-l-rhamnose (73), which is the direct precursor of the rhamnose moieties of the *X. hortorum* pv. *vitians* LPS core and O-antigen (74). Then, the three functional regions of the LPS biosynthesis gene cluster (72) showed different results (Fig. 3b). The LPS 1 region located between *wxcA* (XHV734_4249) and *wxcE* (XHV734_4243) was entirely critical in lettuce and is responsible for the biosynthesis of the water-soluble O-antigen (72). Genes *wzt*, *wxcB*, and *wxcC* (XHV734_4247, -4246, and -4245) were also important for bacterial growth *in vitro*, suggesting that the formation of a functional O-antigen is necessary even in rich medium. Diverse X. campestris pv. *campestris* and *X. oryzae* pv. *oryzae* mutants defective in O-antigen biosynthesis showed reduced or abolished virulence *in planta* (41, 42). The *gmd* gene of LPS region 2 (XHV734_4242) was also critical in lettuce. This region is implied in biosynthesis of the core oligosaccharide of the LPS (72). Finally, none of the LPS 3 region genes, ranging from *wxcK* (XHV734_4236) to *wxcO* (XHV734_4240), was critical. Genes within this last segment probably encode accessory synthesis and transfer of *N*-acetyl-3-amino hexose (72) and contribute to the side branching of the O-antigen (75). Mutating these genes resulted in the production of modified O-antigens in X. campestris pv. *campestris* (75).

Altogether, our findings indicate that certain genes directly implied in biosynthesis of the LPS core oligosaccharide and O-antigen are indispensable for successful bacterial invasion and survival inside lettuce leaves. Tn-seq also showed that some genes involved in biosynthesis of the LPS core and O-antigen were critical for P. syringae and R. solanacearum growth *in planta* (Table 4) (30, 32). Gram-negative bacterial LPS have a general protective role against stresses in diverse environments (76), including oxidative stress (70). They also participate in camouflaging pathogens from the host immune system, although they are also one of the major microbial-associated molecular patterns (MAMPs) recognized by host immunity receptors (77, 78). The long O-antigen of Xylella fastidiosa delays pathogen recognition and the basal immune response in grape (79). If a structurally intact LPS seems to be required for hiding from the lettuce immune system in *X. hortorum* pv. *vitians*, modifications of the O-antigen side branching does not alter this protective role.

**TABLE 4:** *X. hortorum* pv. *vitians* critical genes in lettuce with at least one homolog in other *in planta* Tn-seq/RB-Tn-seq studies conducted on plant-pathogenic bacteria

Locus	Gene	Product	Critical homologous gene(s) in:			
			Pseudomonas syringae ^*a*^	Ralstonia solanacearum ^*b*^	*Agrobacterium fabrum* ^*c*^	Dickeya dadantii ^*d*^
XHV734_0038	*gltD*	Glutamate synthase, 4Fe-4S protein, small subunit			ATU_RS17595	
XHV734_0039	*gltB*	Glutamate synthase, large subunit	Psyr_0411		ATU_RS17605	
XHV734_0130	*tyrB*	Tyrosine aminotransferase, tyrosine-repressible, PLP-dependent		RS_RS05000		
XHV734_0259	*glnA*	Glutamine synthetase			ATU_RS02965	
XHV734_0262		Two-component system sensor protein			ATU_RS07120	Dda3937_01117
XHV734_0263	*glnG*	Fused DNA-binding response regulator, sigma54 interaction protein			ATU_RS07125	Dda3937_01116
XHV734_0278	*secB*	Protein export chaperone		RS_RS01755		
XHV734_0458	*ilvD*	Dihydroxyacid dehydratase	Psyr_0469		ATU_RS09365 ATU_RS18520 ATU_RS14860	
XHV734_0506	*bioB*	Biotin synthase	Psyr_4687			
XHV734_0595	*trpE*	Anthranilate synthase component 1	Psyr_4609	RS_RS14430	ATU_RS11170	
XHV734_0640	*trpD*	Anthranilate phosphoribosyltransferase	Psyr_4580		ATU_RS08260	
XHV734_0642	*trpC*	Indole-3-glycerol phosphate synthase			ATU_RS08265	
XHV734_0670	*purD*	Phosphoribosylglycinamide synthetase phosphoribosylamine-glycine ligase			ATU_RS03175	
XHV734_0671	*purH*	Fused IMP cyclohydrolase; phosphoribosylaminoimidazolecarboxamide formyltransferase			ATU_RS13745	Dda3937_00244
XHV734_0831	*opgH*	Glucans biosynthesis glucosyltransferase H	Psyr_0378	RS_RS14570		Dda3937_03563
XHV734_0943		Transcriptional regulator of *N*-acetylglucosamine utilization, LacI family			ATU_RS21970	
XHV734_0998	*aspC*	Putative aspartate aminotransferase			ATU_RS10725	
XHV734_1041		Type I secretion outer membrane protein, TolC precursor				Dda3937_00726
XHV734_1046	*leuC*	3-Isopropylmalate isomerase subunit, dehydratase component	Psyr_1983		ATU_RS13195	Dda3937_01352
XHV734_1047	*leuD*	3-Isopropylmalate isomerase subunit			ATU_RS13585	
XHV734_1049	*leuB*	3-Isopropylmalate dehydrogenase	Psyr_1985		ATU_RS13590	Dda3937_04404
XHV734_1050	*leuA*	2-Isopropylmalate synthase	Psyr_1257		ATU_RS11045	Dda3937_04301
XHV734_1051	*tdcB*	l-Threonine dehydratase catabolic TdcB			ATU_RS05955	
XHV734_1053	*ilvG*	Acetolactate synthase isozyme 2 large subunit	Psyr_0846		ATU_RS09945	
XHV734_1054	*ilvC*	Ketol-acid reductoisomerase (NADP(+))	Psyr_0848		ATU_RS09860	
XHV734_1096	*hemL*	Glutamate-1-semialdehyde aminotransferase (aminomutase)			ATU_RS22545	
XHV734_1124	*yjgF*	Ketoacid-binding protein				
XHV734_1145	*tktA*	Transketolase 1, thiamin-binding			ATU_RS17355	
XHV734_1167	*gapA*	Glyceraldehyde-3-phosphate dehydrogenase A			ATU_RS17360	
XHV734_1174		Putative fructose-bisphosphate aldolase class 1			ATU_RS17375	
XHV734_1213	*thiG*	Thiamine biosynthesis ThiGH complex subunit	Psyr_4740			
XHV734_1262	*nadE*	Glutamine-dependent NAD(+) synthetase		RS_RS11780		
XHV734_1330		Hypothetical protein			ATU_RS17310	
XHV734_1332	*ruvA*	Component of RuvABC resolvasome, regulatory subunit			ATU_RS17290	
XHV734_1688		Nicotinate-nucleotide adenylyltransferase		RS_RS11005		
XHV734_1761	*trpF*	*N*-(5′-Phosphoribosyl)anthranilate isomerase	Psyr_1663	RS_RS09970	ATU_RS00085	
XHV734_1763	*trpB*	Tryptophan synthase, beta subunit	Psyr_0034	RS_RS09965	ATU_RS00090	
XHV734_1765	*trpA*	Tryptophan synthase alpha chain	Psyr_0033	RS_RS09955	ATU_RS00095	
XHV734_1808	*purK*	N5-carboxyaminoimidazole ribonucleotide synthase			ATU_RS17455	Dda3937_01683
XHV734_1945	*tgt*	tRNA-guanine transglycosylase		RS_RS13575		
XHV734_1977	*gdh*	NAD-specific glutamate dehydrogenase			ATU_RS13460	
XHV734_2148	*purM*	Phosphoribosylaminoimidazole synthetase			ATU_RS05630	Dda3937_02515
XHV734_2154	*sodA*	Superoxide dismutase, Mn			ATU_RS04315	
XHV734_2180		Dihydroorotase			ATU_RS06435	
XHV734_2250		UDP-*N*-acetylmuramyl pentapeptide phosphotransferase/UDP-*N*-acetylglucosamine-1-phosphate transferase			ATU_RS22585	
XHV734_2252	*galU*	UTP-glucose-1-phosphate uridylyltransferase	Psyr_2980		ATU_RS17570	
XHV734_2285	*phaC*	Poly(3-hydroxyalkanoate) polymerase subunit PhaC		RS_RS08220		
XHV734_2316	*zwf*	Glucose-6-phosphate dehydrogenase			ATU_RS02955	
XHV734_2707	*carB*	Carbamoyl-phosphate synthase large subunit				Dda3937_01389
XHV734_2709	*carA*	Carbamoyl phosphate synthetase small subunit, glutamine amidotransferase				Dda3937_01390
XHV734_2726	*serA*	d-3-Phosphoglycerate dehydrogenase	Psyr_4852		ATU_RS17200	
XHV734_2742	*hisF*	Imidazole glycerol phosphate synthase, catalytic subunit with HisH	Psyr_4894		ATU_RS00190	
XHV734_2743	*hisA*	*N*-(5′-Phospho-l-ribosyl-formimino)-5-amino-1-(5′-phosphoribosyl)-4-imidazolecarboxamide isomerase	Psyr_4894		ATU_RS00195	
XHV734_2744	*hisH*	Imidazole glycerol phosphate synthase, glutamine amidotransferase subunit with HisF	Psyr_4896		ATU_RS00200	
XHV734_2745	*hisB*	Fused histidinol-phosphatase; imidazoleglycerol-phosphate dehydratase	Psyr_4897		ATU_RS00210	
XHV734_2747	*hisD*	Bifunctional histidinal dehydrogenase and histidinol dehydrogenase	Psyr_4133		ATU_RS02645	
XHV734_2748	*hisG*	ATP phosphoribosyltransferase	Psyr_4134		ATU_RS03340	
XHV734_2829	*pgi*	Glucose-6-phosphate isomerase	Psyr_0826		ATU_RS01945	
XHV734_2887	*miaA*	Delta(2)-isopentenylpyrophosphate tRNA-adenosine transferase		RS_RS12855	ATU_RS09955	
XHV734_2989	*pyrB*	Aspartate carbamoyltransferase			ATU_RS06440	Dda3937_01284
XHV734_3113	*udg*	UDP-glucose 6-dehydrogenase			ATU_RS12570;	
					ATU_RS19395	
XHV734_3365	*aroA*	3-Phosphoshikimate 1-carboxyvinyltransferase		RS_RS04510		
XHV734_3366	*pheA*	Chorismate mutase/prephenate dehydratase			ATU_RS00480	
XHV734_3367	*serC*	3-Phosphoserine/phosphohydroxythreonine aminotransferase		RS_RS04490		
XHV734_3398	*metXS*	Homoserine *O*-succinyltransferase	Psyr_0474			
XHV734_3771	*clpX*	ATPase and specificity subunit of ClpX-ClpP ATP-dependent serine protease	Psyr_1748	RS_RS08650		
XHV734_3772	*clpP*	Proteolytic subunit of ClpA-ClpP and ClpX-ClpP ATP-dependent serine protease		RS_RS08645		
XHV734_3851	*purF*	Amidophosphoribosyltransferase	Psyr_1668		ATU_RS05325	Dda3937_02099
XHV734_4155	*glyA*	Serine hydroxymethyltransferase	Psyr_4270	RS_RS03670		
XHV734_4196	*purL*	Phosphoribosylformyl-glycineamide synthetase	Psyr_1269			Dda3937_03379
XHV734_4228	*cpsG*	Phosphohexose mutase	Psyr_0219			
XHV734_4229	*cpsB*	Mannose-1-phosphate guanyltransferase			ATU_RS15505	
XHV734_4230	*rmlD*	dTDP-4-dehydrorhamnose reductase subunit, NAD(P)-binding, of dTDP-l-rhamnose synthase		RS_RS03435	ATU_RS21640	
XHV734_4231	*rmlC*	dTDP-4-deoxyrhamnose-3,5-epimerase			ATU_RS22530	
XHV734_4232	*rmlA*	Glucose-1-phosphate thymidylyltransferase		RS_RS03440	ATU_RS21635	
XHV734_4233	*rmlB*	dTDP-glucose 4,6-dehydratase			ATU_RS21645	Dda3937_03924
XHV734_4242	*gmd*	GDP-d-mannose dehydratase, NAD(P)-binding	Psyr_0915			
XHV734_4247	*wzt*	ATP binding component of ABC-transporter	Psyr_0918			
XHV734_4248	*wzm*	Transport permease protein	Psyr_0917			
XHV734_4497		Lipid A core-O-antigen ligase-like enzyme		RS_RS11060		
XHV734_4610	*pyrE*	Orotate phosphoribosyltransferase			ATU_RS01925	Dda3937_03258
XHV734_4646		Flavodoxin reductases (ferredoxin-NADPH reductases) family 1			ATU_RS03585	
XHV734_4897	*tatC*	TatABCE protein translocation system subunit		RS_RS14730		

a Genes required for P. syringae epiphytic and/or apoplastic fitness in bean leaves according to Helmann et al. (30).

b Genes required for R. solanacearum fitness in tomato plants according to Su et al. (32).

c Genes required for *A. fabrum* fitness under at least one condition (i.e., tomato tumors, tomato roots, maize roots, or poplar tumors) according to Torres et al. (33).

d Genes required for *D. dadantii* fitness in chicory leaves according to Royet et al. (29).

### Diversity of resistance mechanisms to the immunity-triggered oxidative burst.

The rapid and localized accumulation of reactive oxygen species (ROS) inside the plant tissues following MAMP recognition by the plant PRRs is referred to as the oxidative burst (13). In general, protection against ROS-induced damage, which includes lipid peroxidation, enzyme inactivation, and nucleic acid degradation, can be achieved in three different ways: (i) prevention of ROS generation, (ii) detoxification of radicals, and (iii) repair of damaged elements (80). We found a set of 10 critical genes in lettuce that we hypothesized to actively participate in protecting the pathogen against the oxidative burst by detoxifying ROS and repairing damaged macromolecules (Fig. 2).

The Mn-superoxide dismutase-encoding gene *sodA* (XHV734_2154) was critical *in planta*. SodA plays a pivotal role in the virulence of several plant-pathogenic bacteria (81). The expression of this gene is induced within the first 3 h of infection in X. campestris pv. *campestris* and *D. dadantii* (82, 83), proving that it acts as a very early protection against the oxidative burst. Interestingly, *sodA* is not directly induced by ROS in X. campestris pv. *campestris*, but by redox cycling agents like plumbagin, a plant quinone with antimicrobial properties (84). Another gene, XHV734_2318, encodes an YgfZ-family folate-dependent protein that might also be involved in plumbagin resistance (85). On the other hand, *phaC* encodes a polymerase producing granules of poly(3-hydroxyalkanoate) (PHA), a polyester that can both serve as a form of carbon storage and can enhance resistance to cold and oxidative stresses in pseudomonads (81).

A variety of genes were implied in the recycling of misfolded proteins and damaged nucleic acids. The *clpX* (XHV734_3771), *clpP* (XHV734_3772), and *ftsH* (XHV734_2889) genes encode the ClpXP serine protease and an ATP-dependent Zn-metalloprotease, respectively. The ClpXP protease recycles misfolded proteins in the cytoplasm (86), and its inactivation in X. campestris pv. *campestris* through *clpX* or *clpP* mutations leads to pleiotropic effects, including increased stress sensitivity (87, 88). The *clpX* and *clpP* genes were also critical *in planta* in an R. solanacearum Tn-seq study (Table 4) (32). Moreover, oxidative stress induces the expression of *ftsH* in Lactobacillus plantarum, and Δ*ftsH* mutants are highly sensitive to various stresses (89). These two proteases are probably mobilized to buffer the damaging effects of the oxidative burst on the bacterial proteome.

Finally, the RNase R-encoding *rnr* gene (XHV734_3096) and the homologous genes of the recombination system *recBCD* (XHV734_5034, -5035, and -5033) were all critical in lettuce. They are probably involved in repairing the damage caused to nucleic acids by immunity-induced ROS production. RNase R is indeed required for the *trans*-translation pathway that directs deficient proteins and transcripts toward degradation while rescuing stalled ribosomes and is important for pathogenesis in many bacterial pathogens (90). This protein is overexpressed in *X. citri* subsp. *citri* during orange tree infection, supporting that it is important for *Xanthomonas* virulence (91). The RecBCD homologous recombination system has been proposed to be the predominant repair mechanism of oxidative stress-induced DNA damage (92–94), probably because it specifically repairs double-stranded breaks—the most deleterious DNA damage caused by oxidative agents (95). However, the *recC* mutant already showed a growth defect in the inoculum, and the *recBCD* genes all displayed low mean read count values *in vitro*. Intriguingly, RecA did not appear to play a role in oxidative stress resistance in *Xanthomonas* (96), which tends to be confirmed by its neutral status in our analysis.

### Specific and common traits of the *in planta* lifestyle unveiled by the comparison with other plant-pathogenic bacteria.

**FIG 4:**
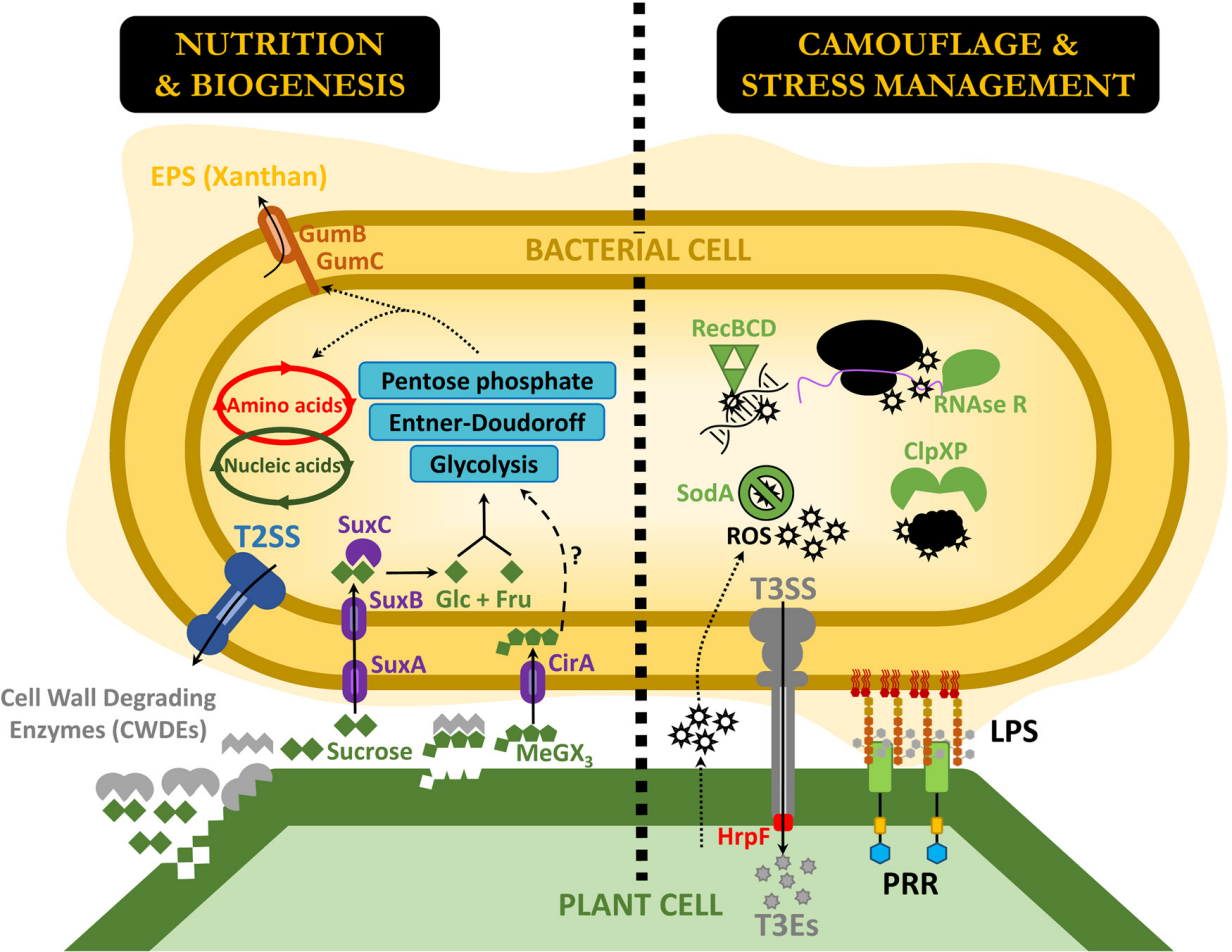
Schematic overview of the critical genes and processes required for lettuce infection by *X. hortorum* pv. *vitians* LM16734 as discussed in this work. Gray objects are known important virulence factors not screened as critical but discussed in the text. Abbreviations: EPS, exopolysaccharide; T2SS, type II secretion system; Glc, glucose; Fru, fructose; MeGX_3_, methylglucuronoxylotriose; ROS, reactive oxygen species; T3SS, type III secretion system; T3Es, type III effectors; LPS, lipopolysaccharide; PRRs, pattern recognition receptors.

The present study presents a first overview of some of the molecular processes required for successful colonization and infection of lettuce leaves by the phytopathogenic bacterium *X. hortorum* pv. *vitians* (Fig. 4). Similar studies have been conducted on a handful of other plant-pathogenic bacteria *in planta* in the past 3 years, allowing us to compare the gene set we identified with those unveiled by other similar studies. We searched for homologs of the genes we identified in the data sets obtained for *D. dadantii* in chicory leaves (29), P. syringae in bean leaves (30), R. solanacearum in tomato roots (32), and A. fabrum under multiple conditions (tomato tumors and roots, maize roots, and poplar tumors) (33).

Eighty-three out of 170 critical genes of *X. hortorum* pv. *vitians* had at least one homolog in one of these other important plant-pathogenic bacterial genes *in planta* (Table 4). More precisely, 57 genes had homologs in *A. fabrum*, 33 in P. syringae, 21 in R. solanacearum, and 17 in *D. dadantii*, even though there was not a single gene found in all 5 studies. The importance of amino acid biosynthesis *in planta* was also true for *A. fabrum* and P. syringae, particularly in the Val/Leu/Ile, Ala/Asp/Glu, Phe/Tyr/Trp, Gly/Ser/Thr, and His biosynthesis pathways, where most of the key genes were critical. Only the genes in the Phe/Tyr/Trp and Gly/Ser/Thr biosynthesis pathways were also critical in R. solanacearum, and only a few genes involved in the Val/Leu/Ile biosynthesis pathway were critical in *D. dadantii*. Most of the genes of the PPP and dDTP-l-rhamnose (a rhamnolipid and LPS component in several bacteria) biosynthesis pathways (97) were critical in both *X. hortorum* pv. *vitians* and *A. fabrum*. Finally, some key genes of the purine and pyrimidine biosynthesis pathways were important too in *A. fabrum* and *D. dadantii*. Overall, it appears that despite a few similarities, each of these 5 pathogens has specific nutritional requirements during its life *in planta*, probably because of the differential nutrient availability in the respective host plants.

Xanthomonas hortorum pv. *vitians* seems to be more dependent than the other pathogens on *de novo* amino acid biosynthesis. Besides the above-cited pathways, the 6 genes *argBCEFG* of the Arg biosynthesis pathway, *thrABC*, *gyaR*, and XHV734_4311 of the Gly/Ser/Thr biosynthesis pathway, and *mtaD* and *mtnBC* of the Cys/Met biosynthesis pathway were also important for fitness in lettuce leaves. Exogenous amino acid availability might therefore be scarcer in the lettuce apoplast than in the tomato, bean, or poplar leaf apoplasts. Mutagenesis of the T2SS was detrimental for *in planta* fitness of *X. hortorum* pv. *vitians*, but not for fitness of R. solanacearum, P. syringae, and *D. dadantii*. Yet, the T2SS is an important virulence factor for these three pathogens (29, 98, 99). Even though an in *trans* complementation effect could account for this phenomenon in the *D. dadantii* Tn-seq study (29), our results suggest that the T2SS may play an unexpectedly important role in the virulence of *X. hortorum* pv. *vitians*. A thorough comparison with other plant-pathogenic bacterial LPSs would be difficult because each bacterium has a specific set of genes involved in LPS biosynthesis (100–102). Yet, the accurate identification of the LPS biosynthesis genomic regions critical for *in planta* fitness of *X. hortorum* pv. *vitians* is also a new finding that strengthens our understanding of the role of this major surface component in *Xanthomonas* pathogenesis. Finally, several genes putatively implied in the degradation and repair of damaged nucleic acids or misfolded proteins (*recBCD*, *rnr*, *ftsH*, and XHV734_2318) were critical only in *X. hortorum* pv. *vitians*, while the *clpXP* protease was also important for *in planta* fitness of P. syringae and R. solanacearum (Table 4). However, different proteases were important *in planta* for the other pathogens: e.g., the *clpAP* protease for *A. fabrum* and *D. dadantii* (29, 33). Although the detrimental consequences of the plant defense mechanisms are relatively similar in all these pathosystems, it seems that each pathogen displays a specific response based on different genes.

More *in planta* Tn-seq studies will undoubtedly be conducted on other plant-pathogenic bacteria in the years to come. The next step will consist of extending the comparison conducted here to see if the gene sets important for *in planta* fitness of plant pathogens belonging to a same genus overlap. A comparison between pathogens conventionally classified as biotrophs or necrotrophs, soilborne or not, or between pathogens infecting a same plant species would also refine our understanding of the complex genetic requirements sustaining these host-pathogen interactions.

## MATERIALS AND METHODS

### Bacterial strains, growth conditions, and Tn-seq library generation.

Xanthomonas hortorum pv. *vitians* LM16734 (=CFBP 8638) was originally isolated from a diseased leaf sample of oakleaf lettuce (Lactuca sativa) collected in the Rhône-Alpes region (France) in 2016 (18). The bacterium was routinely cultured on 1/10^th^ tryptic soy agar (TSA) plates and in 1/10^th^ tryptic soy broth (TSB) at 28°C. The procedure for generating the Tn-seq mutant library was described previously (25). Briefly, mating the recipient *X. hortorum* pv. *vitians* LM16734 strain with a donor Escherichia coli MFDpir/pSamEc strain containing a Himar1-C9 transposase gene yielded approximately 250,000 individual mutant colonies. They were recovered with 1/10^th^ TSB, pooled, and stored in 30% (vol/vol) glycerol vials at −80°C. The resulting mutant library population was estimated to 10^10^ CFU · mL^−1^.

### Monitoring of the bacterial population *in planta.*

The bacterial population was estimated over the course of the infection process in leaf lettuce cv. Météore for 14 days. Plants were cultivated and inoculated as described previously (18). The population size was enumerated 0, 1, 2, 4, 7, 10, and 14 days postinoculation (dpi). At each time point, three infected leaves from three different plants were randomly collected and treated as independent technical replicates. Except for the initial sampling at *t* = 0 dpi, aimed at enumerating the inoculum deposited on the leaves, all other leaf samples were surface sterilized in a 70% ethanol bath for 20 s and rinsed in sterile deionized H_2_O (dH_2_O) to count only the bacteria that had penetrated the leaf tissue. In all cases, 4 disks of 3.14 cm^2^ each were cut with a sterilized 1-cm-diameter punch on either side of the leaf midrib and pooled in 10 mL of sterile dH_2_O. Leaf disks were crushed using a T25 IKA Ultra-turrax disperser (IKA, Staufen im Bresigau, Germany) at full speed. The resulting leaf homogenates were serially diluted and plated onto 1/10^th^ TSA plates supplemented with cycloheximide at 50 μg · mL^−1^ with an easySpiral automated plater (Interscience, Saint-Nom-la-Bretèche, France). The plates were incubated at 28°C for 4 days and enumerated with a Scan 1200 automatic colony counter (Interscience). Bacterial population sizes were expressed in CFU · cm^−2^ of lettuce leaf.

### Inoculation of lettuce with the transposon library.

Two transposon library aliquots were thawed at 4°C overnight and inoculated in two flasks containing 100 mL of 1/10^th^ TSB supplemented with kanamycin at 25 μg · mL^−1^. The cultures were incubated overnight at 28°C under shaking at 120 rpm until they reached an optical density at 600 nm (OD_600_) of 1.4. Then, they were centrifuged at 3,344 × *g* at 4°C for 20 min, and the cell pellets were washed by being resuspended in 25 mL of cold sterile dH_2_O and repeating the centrifugation step. Each cleaned cell pellet was resuspended in sterile dH_2_O and spectrophotometrically adjusted to obtain 400 mL of inoculum at an OD_600_ of 0.2 supplemented with Tween 80 at 0.08% (vol/vol). Half of each inoculum was centrifuged, and the cell pellets were stored at −20°C for DNA extraction of the control condition. The remaining 200 mL of each inoculum was hand-sprayed on the leaves of 30 young lettuce cv. Météore plants per duplicate until runoff. All plants were immediately incubated in an Ineltec environmental chamber (Ineltec France, Vénissieux, France) at 25°C, with an 18-h photoperiod and 90% relative humidity (RH) for 48 h and then 70% RH for the rest of the experiment.

### Recovery of bacterial cells from lettuce leaves.

Diseased lettuce leaves were collected at *t* = 10 dpi, when small water-soaked leaf spot lesions were abundant but not coalescent, and duplicates were treated separately. Leaves were crushed in 500 mL of sterile dH_2_O with a sterilized Moulinex Masterchef 58 electronic blender (SEB Moulinex G S M, Ecully, France) at middle speed for 2 to 3 min until <2-mm rough fragments were obtained. The resulting suspensions were sonicated in a sonication water bath 88155 (Fischer Bioblock Scientific, Illkirch, France) for 15 min to detach bacterial cells from leaf debris. To remove plant debris, the suspensions were successively filtered through 1-, 0.5-, 0.25-, and 0.05-mm-pore-diameter sieves, then twice through 20-μm-pore-diameter coffee filters, and finally vacuum filtered through 10-μm-pore ipPORE track-etched membrane filters (it4ip, Louvain-la-Neuve, Belgium). In the end, approximately 400 mL of filtered bacterial cell suspensions was recovered per duplicate and centrifuged at 10,647 × *g* for 20 min at 4°C, and the cell pellets were temporarily kept at −20°C.

### DNA extraction and library preparation.

Genomic DNA library extraction and preparation were conducted mostly as described previously (25), with technical variations. Briefly, the cell pellets were thawed, and genomic DNA was extracted using a Promega Wizard genomic DNA purification kit (Promega, Madison, WI, USA) and resuspended in 200 μL of Tris-EDTA (pH 7). Fifty micrograms of DNA per sample was digested with the MmeI restriction enzyme (New England Biolabs, Ipswich, MA, USA), and digestion products were purified. Samples were deposited on a 1% (wt/vol) agarose gel, and the 1.3- to 1.8-kb DNA fragments were cut out from the gel and retrieved using a QIAquick gel extraction kit (Qiagen, Hilden, Germany). Then, 300 to 500 ng of each sample was ligated to double-stranded barcoded adapters with the T4 DNA ligase (New England Biolabs). The 20-bp genomic DNA regions flanking the transposon were amplified by 22 cycles of PCRs in 50-μL final volumes. The reaction mixtures contained 2 μL of ligated DNA matrix, 1 U of Q5 DNA polymerase (New England Biolabs), 1× Q5 buffer, 0.2 mM deoxynucleoside triphosphates (dNTPs), and 0.4 μM of each TruSeq primers (Illumina, Inc., San Diego, USA). The PCR products were deposited on a 2% (wt/vol) agarose gel, and the 125-bp bands were extracted using a QIAquick gel extraction kit (Qiagen). Library DNA samples were sent to the I2BC-sequencing platform (I2BC, Gif-sur-Yvette, France) to be sequenced in the “High Output Single Read” 75-bp mode on a NextSeq 500 instrument (Illumina, Inc.).

### Pretreatment of the sequencing read data.

Raw reads were trimmed with CUTADAPT v1.15 (103) to filter those containing the Mariner inverted left repeat (i.e., ACAGGTTGGATGATAAGTCCCCGGTCTT) and remove the adapter sequences. Trimmed data sets were mapped without mismatches to unique TA sites with a modified version of the TPP script from the TRANSIT software suite v2.0.2 (104) on the complete genome sequence of *X. hortorum* pv. *vitians* LM16734 available from the NCBI GenBank database under accession no. GCA_014338485.1. The script generating the prot_table file used by TRANSIT was modified to integrate noncoding RNA (ncRNA) features. *In vitro* library sequencing reads available in the NCBI SRA database under accession no. SRR12067051 and SRR12067052 were retrieved and combined with the newly generated sequencing reads in a single .wig file, and subjected to the “Trimmed Total Reads” (TTR) normalization method in TRANSIT v3.2.1.

### Assessment of the contribution of each gene to fitness *in vitro* and *in planta.*

The contribution of each genetic feature to bacterial fitness was determined both in 1/10^th^ TSB (inoculum) and in lettuce by carrying out pairwise comparisons of (i) the inoculum condition with the previously generated *in vitro* library results (25) and (ii) the lettuce infection condition with the inoculum condition. Reads in the 5% N-terminal and 10% C-terminal portions of the genetic feature were discarded because they could lead to incorrect interpretations, and a LOESS (locally estimated scatterplot smoothing) correction for genome positional bias was applied. The “Resampling” method available in the TRANSIT v3.2.1 suite (105) was used to evaluate the fitness contribution and statistical significance of each genetic feature in the two comparisons, by (i) calculating the difference in the sum of read counts and log_2_ fold change (FC) between the control and experimental conditions, (ii) calculating *P* values using variation of the nonparametric permutation test, and (iii) correcting the *P* values by performing a Benjamini-Hochberg false-discovery rate (FDR) test (106), which yields *q* values expressing the probabilities of false-positive results. The genetic features with a *q* value of ≤0.05 were considered to present a significant fitness difference between the control and experimental conditions, and potential artifactual features were checked manually on the MicroScope platform (https://mage.genoscope.cns.fr) (107). Finally, the circular genome and data mapping were visualized with the R/Shiny application shinyCircos (108).

### Gene annotation, Gene Ontology term enrichment, KEGG pathway mapping, and comparison with other Tn-seq or RB-Tnseq *in planta* experiments.

A mixed annotation strategy was applied to assign Gene Ontology (GO) terms (39) to *X. hortorum* pv. *vitians* LM16734 genes. Protein sequences were extracted from the genome using the cds_extractor script from the bac-genomic-scripts suite (109). Terms were assigned using the web-based tool eggNOG-Mapper v2 (110) against the eggNOG v5.0 database (111) and InterproScan v5.0.0 (112), available on the Galaxy for Genome Annotation (GGA) server (https://annotation.usegalaxy.eu/) (113). Finally, a GO term enrichment analysis was conducted with the “Pathway Enrichment Analysis” method available in TRANSIT, based on the Ontologizer method (114). GO terms with an FDR-adjusted *q* value of ≤0.05 were considered to be significantly enriched in the gene subset. EC numbers, eggNOG orthologs, and Cluster of Orthologous Groups (COG) categories were also assigned with eggNOG-Mapper v2. The Kyoto Encyclopedia of Genes and Genomes (KEGG) (https://www.kegg.jp/) database was used to assign KEGG Orthology (KO) terms to genes with BlastKOALA v2.2 (40). KO terms were mapped to the *X. hortorum* reference pathways (*xhr*) with KEGG Mapper (115). Synteny and gene homology assessment were performed on the MicroScope platform following the guidelines of the MicroScope team annotation (107). Our data were compared with the genes identified as important for *in planta* fitness of P. syringae (30), R. solanacearum (32), Agrobacterium fabrum (33), and *D. dadantii* (29) by searching our *in planta* critical gene set for homologs of these genes using BLASTP. Genes with more than 50% coverage, 30% protein identity and an E value of <10^−5^ were considered homologs as described previously (25), and gene annotations and functions were consistently checked to prevent potential inconsistencies.

### Data availability.

The NCBI GenBank assembly accession number for the complete *X. hortorum* pv. *vitians* LM16734 genome is GCA_014338485.1. Transposon insertion sequencing raw reads have been deposited in the NCBI SRA database under accession no. SRR14385278 to SRR14385281. All data are associated with BioProject no. PRJNA530964 and PRJNA726806.

10.1128/mSystems.01290-21.3 TABLE S2 List of the 170 genes critical in lettuce leaves. Download Table S2, XLSX file, 0.06 MB. Copyright © 2022 Morinière et al. 2022 Morinière et al. https://creativecommons.org/licenses/by/4.0/ This content is distributed under the terms of the Creative Commons Attribution 4.0 International license.

10.1128/mSystems.01290-21.4 TABLE S3 List of the 36 genes critical in 1/10th TSB medium. Download Table S3, XLSX file, 0.02 MB. Copyright © 2022 Morinière et al. 2022 Morinière et al. https://creativecommons.org/licenses/by/4.0/ This content is distributed under the terms of the Creative Commons Attribution 4.0 International license.

10.1128/mSystems.01290-21.5 TABLE S4 List of the 2 growth-advantage genes in lettuce leaves. Download Table S4, XLSX file, 0.01 MB. Copyright © 2022 Morinière et al. 2022 Morinière et al. https://creativecommons.org/licenses/by/4.0/ This content is distributed under the terms of the Creative Commons Attribution 4.0 International license.

## Supplementary Material

Reviewer comments
