# Supplementary figures and images for: A Comprehensive Overview of the Genes and Functions Required for Lettuce Infection by the Hemibiotrophic Phytopathogen Xanthomonas hortorum pv. vitians
Source: mSystems. 2022 Mar 21;7(2):e01290-21. doi: 10.1128/msystems.01290-21 (PMC9040725; doi:10.1128/msystems.01290-21)

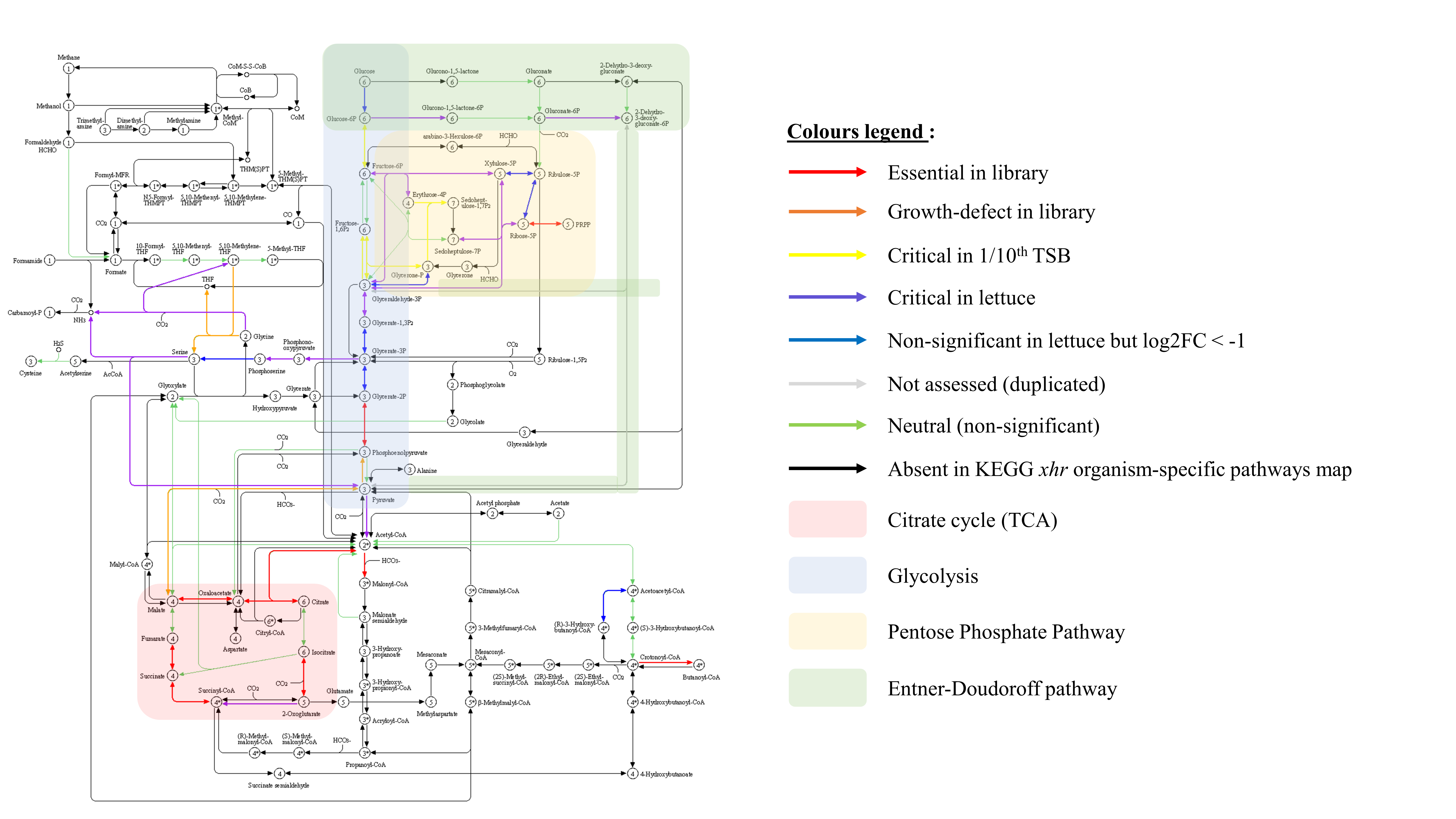

Supplement: FIG S1 [file msystems.01290-21-sf001.tif]
