# Supplementary material for: A Comprehensive Overview of the Genes and Functions Required for Lettuce Infection by the Hemibiotrophic Phytopathogen Xanthomonas hortorum pv. vitians
Source: mSystems. 2022 Mar 21;7(2):e01290-21. doi: 10.1128/msystems.01290-21 (PMC9040725; doi:10.1128/msystems.01290-21)
Supplement: Reviewer comments [file reviewer-comments.pdf]

AMERICAN SOCIETY FOR MICROBIOLOGY | mSystems®

A comprehensive overview of the genes and functions required for lettuce infection by the hemibiotrophic phytopathogen *Xanthomonas hortorum* pv. *vitians*

Lucas Morinière, Laurène Mirabel, Erwan Gueguen, and Frank Bertolla

Corresponding Author(s): Frank Bertolla, Université de Lyon

Review Timeline:

Submission Date:	October 25, 2021
Editorial Decision:	November 29, 2021
Revision Received:	February 4, 2022
Accepted:	February 7, 2022

Editor: Christopher Schadt

Reviewer(s): Disclosure of reviewer identity is with reference to reviewer comments included in decision letter(s). The following individuals involved in review of your submission have agreed to reveal their identity: Steven E. Lindow (Reviewer #1)

Transaction Report:

(Note: With the exception of the correction of typographical or spelling errors that could be a source of ambiguity, letters and reports are not edited. The original formatting of letters and referee reports may not be reflected in this compilation.)

DOI: https://doi.org/10.1128/mSystems.01290-21

November 29, 2021

Dr. Frank Bertolla
Université de Lyon
France

Re: mSystems01290-21 (A comprehensive overview of the genes and functions required for lettuce infection by the hemibiotrophic phytopathogen *Xanthomonas hortorum* pv. *vitians*)

Dear Dr. Frank Bertolla:

Thank you for submitting your manuscript to mSystems. We have completed our review and I am pleased to inform you that, in principle, we expect to accept it for publication in mSystems. However, acceptance will not be final until you have adequately addressed the reviewer comments. Both the reviewers and myself are in agreement that quality of the science is high and in principal worthy of publication in mSystems. However both reviewers note that the discussion in particular could be greatly improved by a more extensive comparison of the results in your study to those using similar approaches in Pseudomonas and other similar organisms. Also I agree with reviewer #1 that the article could greatly benefit from additional english grammar revision and editing. Please pay careful attention to the suggestions of both reviewers in your reply.

Below you will find instructions from the mSystems editorial office and comments generated during the review.

Preparing Revision Guidelines
To submit your modified manuscript, log onto the eJP submission site at https://msystems.msubmit.net/cgi-bin/main.plex. Go to Author Tasks and click the appropriate manuscript title to begin the revision process. The information that you entered when you first submitted the paper will be displayed. Please update the information as necessary. Here are a few examples of required updates that authors must address:

• Point-by-point responses to the issues raised by the reviewers in a file named "Response to Reviewers," NOT IN YOUR COVER LETTER.
• Upload a compare copy of the manuscript (without figures) as a "Marked-Up Manuscript" file.
• Each figure must be uploaded as a separate file, and any multipanel figures must be assembled into one file.
• Manuscript: A .DOC version of the revised manuscript
• Figures: Editable, high-resolution, individual figure files are required at revision, TIFF or EPS files are preferred

For complete guidelines on revision requirements, please see the journal Submission and Review Process requirements at https://journals.asm.org/journal/mSystems/submission-review-process. **Submission of a paper that does not conform to mSystems guidelines will delay acceptance of your manuscript.**

Corresponding authors may join or renew ASM membership to obtain discounts on publication fees. Need to upgrade your membership level? Please contact Customer Service at Service@asmusa.org.

Thank you for submitting your paper to mSystems.

The ASM Journals program strives for constant improvement in our submission and publication process. Please tell us how we can improve your experience by taking this quick Author Survey.

Sincerely,

Christopher Schadt

Editor, mSystems

Journals Department
American Society for Microbiology
1752 N St., NW
Washington, DC 20036
E-mail: peerreview@asmusa.org
Phone: 1-202-942-9338

Reviewer comments:

Reviewer #1 (Comments for the Author):

This manuscript has presented the results of an interesting transposon sequencing study to address those genes that are involved in colonization of lettuce leaves by a Xanthomonas pathogen. The study seems to have been done quite well, and the results clearly implicate the role of over 170 genes in the colonization of lettuce by this pathogen. I was particularly impressed with the thoroughness with which the authors have addressed the bottlenecks associated with transposon sequencing studies. They are very aware of the limitations of the conclusions that would otherwise result had an insufficient number of transposon insertional mutants been introduced into the plants to start the experiments. I found that the first part of the results did a great job of documenting the substantial number of founder mutants in their mutant library. While the manuscript was generally understandable, there are literally hundreds of places where clunky English grammar, inappropriate words, and other issues detract from understanding the manuscript. As such, the manuscript would definitely benefit from extensive editorial revisions to correct these deficiencies in grammar. Since there have now been several similar studies of the use of transposon sequencing, and more recently Tn barseq, to address the contribution of various genes to the colonization of plants, I was disappointed that the authors did not do a better job of trying to summarize the similarity and differences of their results compared to that of other studies of Pseudomonas, and Ralstonia and other taxa that have been interrogated. I kept thinking that some sort of summary table that would have noted the various genes found by the different studies and the extent to which these genes were found in more than one study would have been an excellent way to have shown both the novelty of any genes found here, but especially to better emphasize the common traits that seem to be involved in colonization of different plant species by different plant pathogens. The extensive discussion of the results seem to over and over show how the same or similar genes had been previously shown to be involved in plant host colonization. This seemed quite repetitive after many such examples, and gave me the impression that the study was largely confirmatory of other work. It seemed like there might have been a better way to have summarized the results as discussed above. It also struck me that it might be most effective to have separately and initially discussed those genes that were found to be contributing to fitness that were unique to this study, before moving on to discuss the many genes that had also been found in other bacteria/plant systems to have been involved in fitness. This would have allowed the novelty of anything found in the study to have been emphasized. As it is now, it is very difficult to know to what extent did any genes found here simply confirm that seen in another systems.

Specifics:
Lines 83 through 88. This paragraph seems somewhat disjointed, as the authors talk about leaf surface colonization but then launch into cell wall degrading enzymes and type 2 secretion systems, which would seem to be involved only in the colonization of the apoplast.

Lines 87-88. Again, discussion of biofilms seems out of place at this point.

Line 98. The word should read "benefit"

Line 104 "apparition" seems to be some sort of French word that doesn't make sense in this context. It is not an English word that I have ever seen.

Lines 123 through 124. The use of Tnseq addresses only changes in the growth of bacteria, and would not necessarily directly address "virulence" - which smacks of symptoms etc..

Line 136. The authors repeatedly talk about "TA sites". This needs to be better defined.

Lines 149 through 151. The authors are absolutely correct in noting that the number of generations that their mutant library will experience is an essential factor in the success of the study. They should however better explain this to people who wouldn't necessarily understand why this would be the case.

Lines 154 through 156. I do not think it is accurate to suggest that 5 x 10 to the third bacteria had penetrated the leaves by 24 hours. I suspect that many of these cells reflect the multiplication of a smaller number of cells that had initially invaded the leaf. This needs to be clarified, as this might overestimate the size of the founding population.

Lines 157 through 159. The same issue as the above comment. The number of unique cells that entered compared to how many that might have been grown in the interior of the plant needs to be distinguished.

Line 162. "Virtually" is the wrong word to be using here.

Line 180. It is very clunky to be referring to 1/10th TSB - this should instead be 10% TSB. Also, in this sentence, and throughout the manuscript, the authors talk about "essential genes" -an inappropriate terminology, as such essentiality is usually defined by the fact that mutants in such genes cannot be recovered because they are absolutely required for growth. What they are really suggesting here is that these genes have a significant effect on fitness, but I would not call them "essential".

Lines189 through 200. I have never found that the analysis of GO code frequency is very informative. I did not find this section at all helpful, and would suggest that this paragraph could be eliminated. The GO codes refer to such broad categories of traits that I never found them to be particularly useful.

Lines 218 through 220. More detail should be provided here.

Lines 338 to 339. This introductory sentence seems to jump out of nowhere. And it is unclear how the LPS biosynthesis genes link to anything that were previously discussed.

Line 351. I do not feel it is appropriate to refer to "survival", as they are simply measuring growth, and not survival per se.

Line 359: this is very awkward, because they referred to "growth in the inoculum", whereas they really should be saying "growth of the bacteria in vitro" -- since the inoculum refers to the cells that were applied to the plant, rather than being what was grown per se.

Line 372. These studies addressed growth, and not "survival".

Lines 421 through 424. A good example of a sentence which is definitely in need of improved grammar, as it is hard to understand as currently written.

Lines 429-432. Not only does the sentence not make entire sense, but it definitely needs to be expanded upon to be made clearer what the intention of the sentence was.

Line 434. "Screening" is definitely not the word to be used here.

Line 461. More detail needs to be provided in the methods of inoculation as this is critical to understanding the limitations of this experiment.

Lines 467 through 469. It is not clear what they mean by "die-cut". In addition, it would have been quite helpful to have known the proportion of the total recoverable bacterial population that was epiphytic as a function of time after inoculation of the plants. Initially, in the first day or two after inoculation, I would expect that a high proportion of the bacteria would have been on the surface of the leave, whereas later, more would have been apoplastically located. This detail would provide evidence for whether they were actually looking at apoplastic fitness compared to epithetic fitness.

Reviewer #2 (Comments for the Author):

In this study, the authors used a previously-generated Xanthomonas hortorum transposon library to examine conditionally-important genes for growth in the lettuce apoplast. The resulting lists of conditionally-essential genes in media (TSB) and in planta are broadly useful to comparative studies of plant pathogenic bacteria. Specifically, this manuscript does an excellent job of broadly summarizing the results in the context of functional processes (ie. GO) or metabolic pathways (KEGG) - this is necessary to package these large datasets for the reader. Similarly, as the significance thresholds for these TnSeq analyses are often very stringent, I appreciate the occasional inclusion of genes that presented moderate fitness defects and were likely biologically relevant, even if they did not pass significance cutoffs. Other than some minor comments on the analysis and suggested edits, I do not have major suggestions to improve this manuscript.

Table 1. Would expect a skew in read count per insertion site, and therefore median read count might be more informative than mean.

Since the library was spray inoculated, it is curious that motility/chemotaxis genes were not significant in these results. Depending on the inoculation method, these functions may not be needed, but they usually are required to move into the apoplast. Any hypotheses for this result?

226. Comparison to fungal plant pathogen gene expression is possibly useful, but it would be more informative to include in planta gene expression from more-closely related plant pathogens. In planta gene expression profiles are available for P. syringae and related Xanthomonas spp.

251. High functional redundancy -> what about complementation 'in trans' by neighboring cells?

Minor typos:

113. "techniques"?
121. "it allowed to provide" - awkward
161. "5.6"
231/435. "carbohydrates' metabolism" is used, and might read better simply as "carbohydrate metabolism"

NB: Line numbers in our answers refer to the "Revised Manuscript.docx" document. Line numbers in the automatically-generated merged PDF may differ.

Reviewer #1 (Comments for the Author):

This manuscript has presented the results of an interesting transposon sequencing study to address those genes that are involved in colonization of lettuce leaves by a Xanthomonas pathogen. The study seems to have been done quite well, and the results clearly implicate the role of over 170 genes in the colonization of lettuce by this pathogen. I was particularly impressed with the thoroughness with which the authors have addressed the bottlenecks associated with transposon sequencing studies. They are very aware of the limitations of the conclusions that would otherwise result had an insufficient number of transposon insertional mutants been introduced into the plants to start the experiments. I found that the first part of the results did a great job of documenting the substantial number of founder mutants in their mutant library. While the manuscript was generally understandable, there are literally hundreds of places where clunky English grammar, inappropriate words, and other issues detract from understanding the manuscript. As such, the manuscript would definitely benefit from extensive editorial revisions to correct these deficiencies in grammar. Since there have now been several similar studies of the use of transposon sequencing, and more recently Tn barseq, to address the contribution of various genes to the colonization of plants, I was disappointed that the authors did not do a better job of trying to summarize the similarity and differences of their results compared to that of other studies of Pseudomonas, and Ralstonia and other taxa that have been interrogated. I kept thinking that some sort of summary table that would have noted the various genes found by the different studies and the extent to which these genes were found in more than one study would have been an excellent way to have shown both the novelty of any genes found here, but especially to better emphasize the common traits that seem to be involved in colonization of different plant species by different plant pathogens. The extensive discussion of the results seem to over and over show how the same or similar genes had been previously shown to be involved in plant host colonization. This seemed quite repetitive after many such examples, and gave me the impression that the study was largely confirmatory of other work. It seemed like there might have been a better way to have summarized the results as discussed above. It also struck me that it might be most effective to have separately and initially discussed those genes that were found to be contributing to fitness that were unique to this study, before moving on to discuss the many genes that had also been found in other bacteria/plant systems to have been involved in fitness. This would have allowed the novelty of anything found in the study to have been emphasized. As it is now, it is very difficult to know to what extent did any genes found here simply confirm that seen in another systems.

A: The manuscript has been since corrected by a professional English proofreader (see Acknowledgments section **l. 630-631**). A comparison of the genes we identified as important for the fitness of *X. hortorum* pv. *vitians* in lettuce with those identified in previous *in planta* Tn-seq or RB-Tnseq studies conducted on *Pseudomonas syringae* (1), *Ralstonia solanacearum* (2), *Agrobacterium fabrum* (3) and *Dickeya dadantii* (4) was performed (see Methods section l. **609-614**). Results have been included in **Table S2** and genes of *X. hortorum* pv. *vitians* with at least one homolog in any of these other pathogens have been summarized in a new table (**Table 4**). A section highlighting the common and specific traits unveiled by this comparison has been added to replace the conclusion (l. **435-489**).

Specifics:

Q: Lines 83 through 88. This paragraph seems somewhat disjointed, as the authors talk about leaf surface colonization but then launch into cell wall degrading enzymes and type 2 secretion systems, which would seem to be involved only in the colonization of the apoplast.

A: The sentence has been modified to focus on the role of TonB-dependent transporters in the epiphytic lifestyle (**l. 68-72**).

Q: Lines 87-88. Again, discussion of biofilms seems out of place at this point.
A: Biofilm formation is yet an important feature for the epiphytic fitness of *Xanthomonas* populations, as demonstrated for *X. citri* pv. *citri* (5), *X. fuscans* subsp. *fuscans* (6) or *X. vesicatoria* (7).

Q: Line 98. The word should read "benefit"
A: Corrected (**l. 82**).

Q: Line 104 "apparition" seems to be some sort of French word that doesn't make sense in this context. It is not an English word that I have ever seen.
A: Corrected (**l. 88**).

Q: Lines 123 through 124. The use of Tnseq addresses only changes in the growth of bacteria, and would not necessarily directly address "virulence" - which smacks of symptoms etc..
A: Word "virulence" has been deleted from the sentence (**l. 107**).

Q: Line 136. The authors repeatedly talk about "TA sites". This needs to be better defined.
A: "TA sites" was changed to "TA dinucleotide sites" (**l. 120**). Himar1 transposons like the one used in this study insert specifically at TA dinucleotide in the genome.

Q: Lines 149 through 151. The authors are absolutely correct in noting that the number of generations that their mutant library will experience is an essential factor in the success of the study. They should however better explain this to people who wouldn't necessarily understand why this would be the case.
A: Two sentences have been added to clarify the importance of considering the number of generations (**l. 135-137**).

Q; Lines 154 through 156. I do not think it is accurate to suggest that 5 x 10 to the third bacteria had penetrated the leaves by 24 hours. I suspect that many of these cells reflect the multiplication of a smaller number of cells that had initially invaded the leaf. This needs to be clarified, as this might overestimate the size of the founding population.
A; The paragraph has been modified to qualify our estimations about the size of the founding population (**l. 138-155**).

Q; Lines 157 through 159. The same issue as the above comment. The number of unique cells that entered compared to how many that might have been grown in the interior of the plant needs to be distinguished.
A: Cf. previous answer.

Q: Line 162. "Virtually" is the wrong word to be using here.
A: Replaced by "theoretically" (**l. 152**).

Q: Line 180. It is very clunky to be referring to 1/10th TSB - this should instead be 10% TSB. Also, in this sentence, and throughout the manuscript, the authors talk about "essential genes" -an inappropriate terminology, as such essentiality is usually defined by the fact that mutants in such genes cannot be recovered because they are absolutely required for growth. What they are really suggesting here is that these genes have a significant effect on fitness, but I would not call them "essential".

A; Concerning the replacement of “1/10th TSB” by “10% TSB”, both writings coexist in the scientific literature. Since we used the expressions “1/10th TSA” and “1/10th TSB” in our previous articles (8, 9), it is our opinion that the same writing should be used here for consistency.
Regarding the second remark about “essential genes”, terminology was changed to “critical genes” or “conditionally-essential genes” throughout the manuscript.

Q: Lines189 through 200. I have never found that the analysis of GO code frequency is very informative. I did not find this section at all helpful, and would suggest that this paragraph could be eliminated. The GO codes refer to such broad categories of traits that I never found them to be particularly useful.
A: The GO term enrichment analysis is part of our workflow and contributes to define the general functional processes that are critical for lettuce infection. Moreover, reviewer #2 stated that the GO term analysis was pertinent in their opinion.

Q: Lines 218 through 220. More detail should be provided here.
A: A sentence briefly detailing the results of a random screen conducted in *X. campestris* pv. *campestris* was added (l.**212-215**).

Q: Lines 338 to 339. This introductory sentence seems to jump out of nowhere. And it is unclear how the LPS biosynthesis genes link to anything that were previously discussed.
A: This sentence is directly linked to the previous paragraph which discuss genes associated with cellular polysaccharides metabolism. Lines **339-341 : “**As a result, two genes of the *gum* gene cluster encoding the synthesis of the EPS xanthan and 19 genes involved in the biosynthesis and assembly of cell-surface LPS, clustered in 3 distinct genomic regions, were identified (**Fig. 2**).”

Q: Line 351. I do not feel it is appropriate to refer to "survival", as they are simply measuring growth, and not survival per se.
A: Modified to “growth” (**l. 362**).

Q: Line 359: this is very awkward, because they referred to "growth in the inoculum", whereas they really should be saying "growth of the bacteria in vitro" -- since the inoculum refers to the cells that were applied to the plant, rather than being what was grown per se.
A: Changed to “bacterial growth *in vitro*” (**l. 369**).

Q: Line 372. These studies addressed growth, and not "survival".
A: Modified to “growth” (**l. 382**).

Q: Lines 421 through 424. A good example of a sentence which is definitely in need of improved grammar, as it is hard to understand as currently written.
A: The sentence was modified to : “However, the *recC* mutant was already growth-defect in the inoculum, and the *recBCD* genes all displayed low mean read count values *in vitro*.” (**l. 431-432**)

Q: Lines 429-432. Not only does the sentence not make entire sense, but it definitely needs to be expanded upon to be made clearer what the intention of the sentence was.
A: The conclusion was replaced by a new section comparing our results to other (RB)Tnseq studies conducted on a few plant-pathogenic bacteria (**l. 435-489**).

Q: Line 434. "Screening" is definitely not the word to be used here.
A: Cf. previous answer.

Q: Line 461. More detail needs to be provided in the methods of inoculation as this is critical to understanding the limitations of this experiment.
A: The inoculation procedure is detailed in the next section of the Methods section ("Inoculation of lettuce with the transposon library", l. **517-530**) and in our previous article (8). Thus, it would be repetitive to detail our inoculation procedure twice.

Q: Lines 467 through 469. It is not clear what they mean by "die-cut". In addition, it would have been quite helpful to have known the proportion of the total recoverable bacterial population that was epiphytic as a function of time after inoculation of the plants. Initially, in the first day or two after inoculation, I would expect that a high proportion of the bacteria would have been on the surface of the leave, whereas later, more would have been apoplastically located. This detail would provide evidence for whether they were actually looking at apoplastic fitness compared to epithetic fitness.
A: "Die-cut" was replaced by "cut" (**l. 510**). Knowledge of the total bacterial population would have been informative since we did not surface sterilize the leaves during the Tn-seq experiment. However, the monitoring of bacterial growth *in planta* performed before the Tn-seq experiment showed that the apoplastic population reached a high density two days after inoculation (**Fig. 1a**). Since we recovered the mutant library at 10 DPI, we expect that the majority of cells recovered at this stage were apoplastic. Moreover, Helmann *et al.* (1) found that out of the 30 important genes for the epiphytic fitness of *P. syringae* on bean leaves, 22 were also important for apoplastic growth. A minority of the important genes or the *in planta* fitness of *X. hortorum* pv. *vitians* could thus be involved in the epiphytic lifestyle rather than the apoplastic one. That is why we consistently refered to "important genes for growth in lettuce / *in planta*" throughout the article rather than "important genes for apolastic growth".

Reviewer #2 (Comments for the Author):

In this study, the authors used a previously-generated Xanthomonas hortorum transposon library to examine conditionally-important genes for growth in the lettuce apoplast. The resulting lists of conditionally-essential genes in media (TSB) and in planta are broadly useful to comparative studies of plant pathogenic bacteria. Specifically, this manuscript does an excellent job of broadly summarizing the results in the context of functional processes (ie. GO) or metabolic pathways (KEGG) - this is necessary to package these large datasets for the reader. Similarly, as the significance thresholds for these TnSeq analyses are often very stringent, I appreciate the occasional inclusion of genes that presented moderate fitness defects and were likely biologically relevant, even if they did not pass significance cutoffs. Other than some minor comments on the analysis and suggested edits, I do not have major suggestions to improve this manuscript.

Q: Table 1. Would expect a skew in read count per insertion site, and therefore median read count might be more informative than mean.
A: Table 1 has been modified to display the median read count over non-zero TA sites (**l. 1024**).

Q: Since the library was spray inoculated, it is curious that motility/chemotaxis genes were not significant in these results. Depending on the inoculation method, these functions may not be needed, but they usually are required to move into the apoplast. Any hypotheses for this result?
A: Cf. answer below.

Q: 226. Comparison to fungal plant pathogen gene expression is possibly useful, but it would be more informative to include in planta gene expression from more-closely related plant pathogens. In planta gene expression profiles are available for P. syringae and related Xanthomonas spp.

A: The comparison to the transcriptomic analyses of *Phytophtora infestans* and *Pythium ultimum* was replaced by a comparison with the *in planta* transcriptomes of *X. campestris* pv. *campestris*, *X. oryzae* pv. *oryzicola* and *P. syringae* (**l. 219-241**). This new paragraph focuses on the absence of chemotaxis and motility genes in our Tn-seq screening and uses these transcriptomic data to reconsider the importance of these genes in plant-pathogenic *Xanthomonas* and *Pseudomonas* species.

Q: 251. High functional redundancy -> what about complementation 'in trans' by neighboring cells?
A: Sentence changed to “These genes were not essential in our analysis, either because of their high functional redundancy or of potential complementation *in trans* by neighboring cells.” (**l. 261-263**)

Minor typos:

Q: 113. "techniques"?
A: corrected to “techniques” (**l. 97**)
Q: 121. "it allowed to provide" – awkward
A: corrected to “it provided” (**l. 105**)
Q: 161. "5.6"
A: corrected to “5.6” (**l. 151**)
Q: 231/435. "carbohydrates' metabolism" is used, and might read better simply as "carbohydrate metabolism"
A: corrected to “carbohydrate metabolism” (**l. 243**)

Literature

1. Helmann TC, Deutschbauer AM, Lindow SE. 2019. Genome-wide identification of *Pseudomonas syringae* genes required for fitness during colonization of the leaf surface and apoplast. PNAS 116:18900–18910.
2. Su Y, Xu Y, Liang H, Yuan G, Wu X, Zheng D. Genome-wide identification of *Ralstonia solanacearum* genes required for survival in tomato plants. mSystems 6:e00838-21.
3. Torres M, Jiquel A, Jeanne E, Naquin D, Dessaux Y, Faure D. 2022. *Agrobacterium tumefaciens* fitness genes involved in the colonization of plant tumors and roots. New Phytologist 233:905–918.
4. Royet K, Parisot N, Rodrigue A, Gueguen E, Condemine G. 2019. Identification by Tn-seq of *Dickeya dadantii* genes required for survival in chicory plants. Molecular Plant Pathology 20:287–306.
5. Rigano LA, Siciliano F, Enrique R, Sendín L, Filippone P, Torres PS, Qüesta J, Dow JM, Castagnaro AP, Vojnov AA, Marano MR. 2007. Biofilm formation, epiphytic fitness, and canker development in *Xanthomonas axonopodis* pv. *citri*. MPMI 20:1222–1230.
6. Darrasse A, Carrère S, Barbe V, Boureau T, Arrieta-Ortiz ML, Bonneau S, Briand M, Brin C, Cociancich S, Durand K, Fouteau S, Gagnevin L, Guérin F, Guy E, Indiana A, Koebnik R, Lauber E, Munoz A, Noël LD, Pieretti I, Poussier S, Pruvost O, Robène-Soustrade I, Rott P, Royer M, Serres-Giardi L, Szurek B, van Sluys M-A, Verdier V, Vernière C, Arlat M, Manceau C, Jacques M-A. 2013. Genome sequence of *Xanthomonas fuscans* subsp. *fuscans* strain 4834-R reveals that flagellar motility is not a general feature of xanthomonads. 1. BMC Genomics 14:1–30.
7. Felipe V, Romero AM, Montecchia MS, Vojnov AA, Bianco MI, Yaryura PM. 2018. *Xanthomonas vesicatoria* virulence factors involved in early stages of bacterial spot development in tomato. Plant Pathology 67:1936–1943.
8. Morinière L, Burlet A, Rosenthal ER, Nesme X, Portier P, Bull CT, Lavire C, Fischer-Le Saux M, Bertolla F. 2020. Clarifying the taxonomy of the causal agent of bacterial leaf spot of lettuce through a polyphasic approach reveals that *Xanthomonas cynarae* Trébaol et al. 2000 emend. Timilsina et al. 2019 is a later heterotypic synonym of *Xanthomonas hortorum* Vauterin et al. 1995. Systematic and Applied Microbiology 43:126087.
9. Morinière L, Lecomte S, Gueguen E, Bertolla F. 2021. *In vitro* exploration of the *Xanthomonas hortorum* pv. *vitians* genome using transposon insertion sequencing and comparative genomics to discriminate between core and contextual essential genes. Microbial Genomics 7:000546.

February 7, 2022

Dr. Frank Bertolla
Université de Lyon
Villeurbanne
France

Re: mSystems01290-21R1 (A comprehensive overview of the genes and functions required for lettuce infection by the hemibiotrophic phytopathogen *Xanthomonas hortorum* pv. *vitians*)

Dear Dr. Frank Bertolla:

Thank you for submitting your revisions in response to the previous round of review. You have documented these changes well and it appears you have addressed all significant concerns with your revisions and new analyses. Your manuscript has now been accepted, and I am forwarding it to the ASM Journals Department for publication. For your reference, ASM Journals' address is given below.

Before the article can be scheduled for publication, your manuscript will be checked by the mSystems production staff to make sure that all elements meet the technical requirements for publication. They will contact you if anything needs to be revised before copyediting and production can begin. Otherwise, you will be notified when your proofs are ready to be viewed.

ASM policy requires that data be available to the public upon online posting of the article, so please verify all links to sequence records, if present, and make sure that each number retrieves the full record of the data. If a new accession number is not linked or a link is broken, provide production staff with the correct URL for the record. If the accession numbers for new data are not publicly accessible before the expected online posting of the article, publication of your article may be delayed; please contact the ASM production staff immediately with the expected release date.

As an open-access publication, mSystems receives no financial support from paid subscriptions and depends on authors' prompt payment of publication fees as soon as their articles are accepted.

Publication Fees:
You will be contacted separately about payment when the proofs are issued; please follow the instructions in that e-mail. Arrangements for payment must be made before your article is published. For a complete list of **Publication Fees**, including supplemental material costs, please visit our website.

Corresponding authors may join or renew ASM membership to obtain discounts on publication fees. Need to upgrade your membership level? Please contact Customer Service at Service@asmusa.org.

For mSystems research articles, you are welcome to submit a short author video for your recently accepted paper. Videos are normally 1 minute long and are a great opportunity for junior authors to get greater exposure. Importantly, this video will not hold up the publication of your paper, and you can submit it at any time.

Details of the video are:

· Minimum resolution of 1280 x 720
· .mov or .mp4. video format
· Provide video in the highest quality possible, but do not exceed 1080p
· Provide a still/profile picture that is 640 (w) x 720 (h) max
· Provide the script that was used

We recognize that the video files can become quite large, and so to avoid quality loss ASM suggests sending the video file via https://www.wetransfer.com/. When you have a final version of the video and the still ready to share, please send it to mSystems staff at msystemsjournal@msubmit.net.

For mSystems research articles, if you would like to submit an image for consideration as the Featured Image for an issue, please contact mSystems staff at msystemsjournal@msubmit.net.

Thank you for submitting your paper to mSystems.

Sincerely,

Christopher Schadt
Editor, mSystems

Journals Department
American Society for Microbiology
1752 N St., NW
Washington, DC 20036
E-mail: peerreview@asmusa.org
Phone: (202) 737-3600

Table S2: Accept
Table S1: Accept
Figure S1: Accept
Table S3: Accept
Table S4: Accept
